# The Shape of an Auxin Pulse, and What It Tells Us about the Transport Mechanism

**DOI:** 10.1371/journal.pcbi.1004487

**Published:** 2015-10-20

**Authors:** Graeme Mitchison

**Affiliations:** Sainsbury Laboratory, Cambridge University, Cambridge, United Kingdom; University of Michigan, UNITED STATES

## Abstract

Auxin underlies many processes in plant development and physiology, and this makes it of prime importance to understand its movements through plant tissues. In stems and coleoptiles, classic experiments showed that the peak region of a pulse of radio-labelled auxin moves at a roughly constant velocity down a stem or coleoptile segment. As the pulse moves it becomes broader, at a roughly constant rate. It is shown here that this ‘spreading rate’ is larger than can be accounted for by a single channel model, but can be explained by coupling of channels with differing polar transport rates. An extreme case is where strongly polar channels are coupled to completely apolar channels, in which case auxin in the apolar part is ‘dragged along’ by the polar part in a somewhat diffuse distribution. The behaviour of this model is explored, together with others that can account for the experimentally observed spreading rates. It is also shown that saturation of carriers involved in lateral transport can explain the characteristic shape of pulses that result from uptake of large amounts of auxin.

## Author Summary

Auxin is one of the most important signalling molecules in plants. It is a key player in the development of veins and plant organs, and in responses that the plant makes to light and gravity. Yet we have a rather limited understanding of how auxin moves around plant tissues. I show here that a classic experiment, first carried out almost 50 years ago, has more information hidden in it than one might suppose. In this experiment, one studies how a pulse of radioactive auxin moves down a stem. The first lesson is that the peak of the pulse moves with a well-defined velocity, usually around 1 cm per hour. It has also been observed that the pulse spreads out as it moves, which is perhaps unsurprising, since any source of noise would be expected to have this effect. I show, though, that it is not easy to account for the degree of spreading in terms of noise. Instead, I propose that auxin travels down stems via several, probably many, coupled channels with differing transport rates. This gives one some insight into how plants manage their auxin signalling and puts some constraints on the underlying parameters.

## Introduction

Auxin is the key integrating signal in plants [1, 2], and the dynamics of its movement within the plant is crucial to understanding development [3–9] and a multitude of physiological responses [10–12]. A traditional way to observe these dynamics in stems or coleoptiles is to apply a pulse of radioactively-labelled auxin at one end of a segment of the tissue, allow it to be transported for some period of time, and then cut up the segment into small pieces and measure the amount of label in each piece [13–15]. The profiles one obtains this way allow one to make some inferences about the underlying mechanism [14, 16].

One parameter that is often measured is the velocity of the pulse. It provides information about the underlying permeabilities and diffusion constants of the transport mechanism [16, 17]. I show here that one can also measure the rate at which the pulse spreads out, and that the values one obtains imply further constraints on the underlying mechanism. These constraints lead one to reject single channel models and to consider a variety of ways in which multiple auxin channels may be coupled together. (Here the term ‘channel’ will be used for a file of cells specialised for auxin transport, though later we also consider intracellular compartments as potential channels. Proteins that move auxin in or out of cells will be called transporters, more specifically importers or exporters.)

Models of auxin transport with many channels have certainly been considered before; in particular, the flow and counterflow pattern of auxin in the root has been modelled and applied to gravitropism and growth control [18–20]. However, the aim here is somewhat different, namely, to use the shape of an auxin pulse to diagnose properties of the stem transport system.

## Results

### The shape of an auxin pulse

The classic experiments on auxin transport in coleoptiles [13] show a pulse moving basipetally and broadening as it goes. One would like to measure both the velocity, *v*, of this pulse and what will be called the *spreading rate*, denoted by *ρ*, which is defined to be the rate at which the variance of the pulse increases. It is not always straightforward to measure the velocity, because there are typically several components to the auxin transport profile [14, 21]. In particular, there are fixed or slower-moving components that trail behind the main pulse and may obscure its shape. These features cause even greater difficulties when measuring the variance, as small components far from the peak can cause a large increase in the variance.

**Fig 1:**
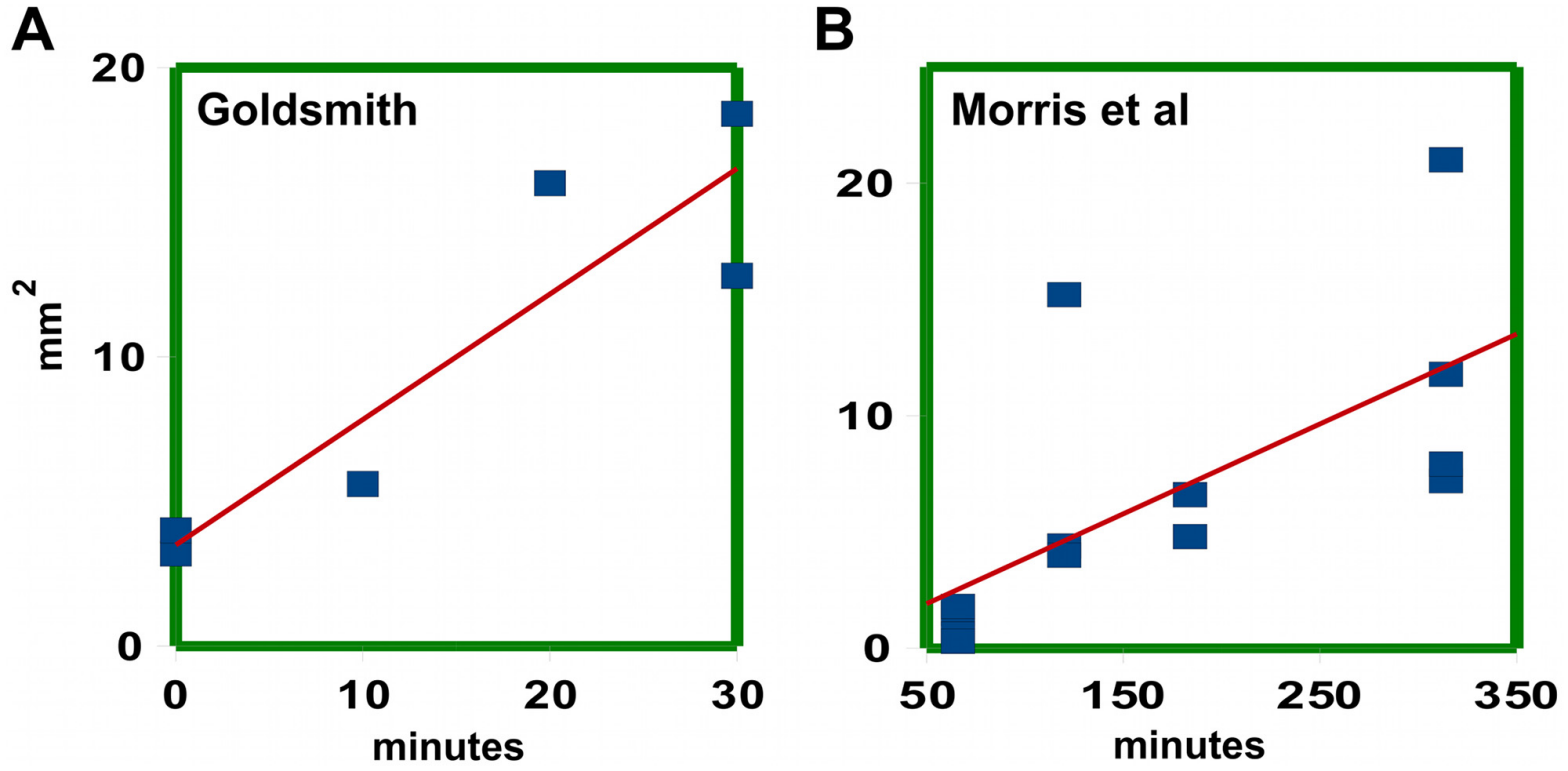
Linear fit of variance plotted against time. A: Using data from Goldsmith’s Fig 1A–1D, 1G and 1H in [13], variances were obtained by least squares. B: Variances obtained by maximal fit, using data from individual segments underlying the averaged curves in Fig 4 of Morris et al. [15].

One strategy would be to try to fit the data to a composite model that attempts to explain all the features in the profile. An alternative strategy, taken here, is to try to isolate the main peak, separating it from trailing or fast moving components. There are reasons to expect the shape of a pulse due to a single channel to be gaussian in form (S1 Text). The data were therefore fitted to a gaussian using least-squares, in the hope that this would pick out the main peak. This works quite well for the data from Goldsmith [13]; see S1 Fig. Given these fits, one can plot the mean and variance against time to obtain estimates for the velocity and spreading rate; see Table 1 and Fig 1A.

**Table 1:** Velocities and spreading rates for various data sets.

Data Set	method	velocity *v*	spreading rate *ρ*	*q*/*p*
Goldsmith 1967	least squares	10.7 ± 1.6	26.0 ± 6.0	11.7
”	maximal fit	11.8 ± 1.8	16.2 ± 5.2	6.4
Morris et al. 2005	by eye	8.8 ± 1.1	10.1 ± 1.8	5.2
”	maximal fit	9.0 ± 0.7	9.8 ± 3.1	4.9

Velocity, in mm/hr, and spreading rate, in mm^2^/hr, for various data sets. The ratio q/p, of diffusion-like to polar permeabilities, is calculated using Eqs (2) and (3), assuming a cell length L of 100 microns.

Goldsmith’s data points represent averages of several coleoptile segments, four for the zero time point and two for all other time points. This introduces the danger that the spreading rate may be over-estimated, since averaging two pulses moving at slightly different velocities will give rise to an artifactual increase of variance with time as the pulses separate. The overestimate will probably not be very large, being of the order of the variance of the velocity distribution. However, one can entirely avoid this objection by using the counts from individual segments. Here we used the single-segment data (S1 Data) underlying the averaged curves in Fig 4 in Morris et al. [15]. These were kindly provided by Dr Morris.

The raw data are of course noisier, and the problem is to determine the shape of the main peak in a reliable manner. Least squares performs poorly in this noisier setting (S2A Fig), as it attempts to fit the entire profile. However, one can modify the fitting algorithm so that it more effectively selects the peak region and ignores flanking regions. We call this procedure *maximal fit* (S1 Text and S2B Fig). One can also select the peak region by eye, trying to err towards excess width at short times and narrower regions at long times, so as to give a conservative estimate of the spreading rate. There is good agreement between this procedure and the maximal fit algorithm at long time intervals (S3 Fig); at shorter times the match is less good (S4 Fig) because of edge effects. Nonetheless, the estimates of spreading rate obtained by the two methods agree well, Table 1, and the estimates of velocity and spreading rate obtained from Goldsmith’s data by least squares and maximal fit also agree reasonably well, within their error ranges.

The conclusion of this section is that it is possible to measure both the velocity and the spreading rate of the main peak of an auxin pulse, though the spreading rate is more vulnerable to noise and questions of interpretation.

### A simple model

Next we ask what the velocity and spreading rate can tell us about the underlying transport mechanism. Consider first a simple model consisting of a row of cells, where auxin is assumed to reach a uniform distribution rapidly inside cells and the flux *ϕ* between cell *n*−1 and cell *n* takes the form

$$\begin{array}{c} \phi =pa_{n-1}+q\left(a_{n-1}-a_{n}\right), \end{array}$$ (1)

where *a*_*i*_ denotes the auxin concentration in cell *i*. Thus there is a polar component to auxin transport between cells given by the permeability *p* and a symmetric, diffusion-like component given by the permeability *q*. In the next section it will be shown how *p* and *q* may relate to known auxin carriers. Note that a non-zero *q* can be produced by combinations of polar carriers, and does not necessarily imply that there is an underlying physical diffusive coupling.

The simple model is appropriate where the cells are small enough for intracellular diffusion to be rapid (e.g. 10 microns or less in length). For instance, an implicit model of this kind underlies treatments of canalization or up-the-gradient mechanisms near to the apical meristem. It is probably not such a good model for the auxin pulse experiments we are considering, since the relevant cells, e.g. the xylem parenchyma, are long enough (e.g. 100 microns) for intracellular diffusion times to be significant. Nonetheless, it serves as a useful warm-up exercise.

One can show (S2 Text) that the velocity, *v*, and the spreading rate, *ρ*, are given by

v = p, (2)

ρ = L(p+2q), (3)

where *L* is the cell length. Given an estimate of cell length L, knowing the velocity and the spreading rate fully characterises the model. In fact, the pulse at time *t* is well approximated by a gaussian of mean *vt* and variance *ρt*; see S2 Text. Fitting the data with a gaussian, as was done in the last section, therefore has some theoretical justification.

The data in Table 1 allow one to calculate *p* and *q*. The velocity gives the permeability *p*, and taking L = 100 microns and using Eq (3) gives q. Instead of giving *q* itself in the Table, we give the ratio *q*/*p*, which is small when the movement of auxin is essentially polar and large when the diffusion-like coupling between cells dominates. As can be seen, the inferred value of *q*/*p* is large in all the data sets.

A version of this model has been used by Renton et al. [22] (their model I). The units in their version are not individual cells but 2 mm segments of stem (the length of the pieces into which the stem is subdivided for counting labelled auxin). They assume that these segments are coupled by a completely polar flux; i.e. *q* = 0. They are able to fit the peak region in their data by taking p = 9 mm/hr. Putting L = 2 mm, p = 9 mm/hr and q = 0 in Eq (3) gives *ρ* = 18 mm^2^/hr. This is larger than our estimate in Table 1, which could be due to the fact that they use averaged data and different fitting criteria.

There is a caveat here. In the above model, and in other models where a piece of tissue is used as the computational unit, e.g. the ‘metamers’ in [23], this computational strategy may offer a gain in speed and simplicity. But care is needed in interpreting the results at a cellular level. In the above model, we can ask what parameter values would be needed to achieve the same velocity and spreading rate with cells as the computational units instead of 2 mm pieces. Taking L = 100 microns, with a velocity of 9 mm/hr and spreading rate of 18 mm^2^/hr (as calculated above), Eq (3) gives 18 = *Lp*(1 + 2*q*/*p*) = 0.1 × 9 × (1 + 2*q*/*p*), or *q*/*p* = 9.5. Thus the completely polar segment-based model translates into a model at the cell level where the diffusive component is much larger than the polar component.

We now turn to the key question: is this simple model plausible? We have already pointed out that one needs to take account of intracellular diffusion in a realistic model. Another potentially unrealistic feature of the model is the dominance of diffusive coupling over polar transport between cells, implied by the values of q/p in Table 1. Yet the strongly polar localization of the auxin transporter PIN1 [24] in what are presumed to be the principal auxin-transporting tissues of the stem suggests a high overall polarity of transport. We consider next whether this view is justified.

### Auxin transporters and the flux between cells

Several families of auxin importers and exporters assist the movement of auxin between plant cells. One family of exporters consists of the PIN-formed family (PINs) [25, 26]. Some PINs, e.g. PIN5 [27], PIN6 [28] and PIN8 [29] are involved in movement between compartments in cells, and will be considered later. The remaining PINs are associated with the plasma membrane, and often show polar localisation within cells, e.g. a basal location of PIN1 in the stem [24], or a more complex pattern of polarity at the shoot apex [30], or a basal localization of PIN2 in epidermal root cells [31], or a gravitropic movement of PIN3 to the bottom side of *Arabidopsis* hypocotyl endodermal cells [12].

Another family of exporters consists of the ABCB transporters (also known as P-glycoproteins, or PGPs) [32, 33]. These are generally uniformly distributed over the plasma membrane, but can sometimes be polar. Thus ABCB1 is known to be uniformly distributed at the shoot and root apices but polarly localized in the mature root cortex and endodermis [32], where it is found in the basal membrane, as are PINs [34].

Some ABCBs, e.g. ABCB21 [35] and ABCB4 [36], may act both as exporters and importers, according to the balance of internal and external auxin concentrations. However, the predominant class of importers is the AUX/LAX family [26, 37, 38]. In general, these too seem to be uniformly distributed in the plasma membrane, though in one special case, the protophloem of *Arabidopsis* roots, AUX1 is asymetrically distributed [38].

Auxin can also be imported passively, without a specific protein channel, because of the lipid solubility of the protonated acid and the fact that the apoplast is acidic (pH typically 4.5 to 5). As the pK of the carboxyl group of auxin is 4.7, about half the auxin in the apoplast will be protonated, and therefore able to enter a cell fairly easily. This is one of the twin pillars of the chemiosmotic theory [39–42], the other being that auxin inside a cell, where the pH is higher (cytoplasmic pH 7.2 [43]), will pass through the membrane far less readily, being about 99.7% in the charged anion form. Thus auxin is in effect trapped inside cells, and exporters are required for intercellular auxin movement, whereas importers would seem less necessary. However, recent measurements of the permeability of the cell membrane to protonated auxin are about two orders of magnitude lower than those available when the theory was formulated. For instance Rutschow et al. [44] find values for *P*_*IAAH*_ close to those of Delbarre et al. [45], at around 4 × 10^−5^ cm/sec, whereas older measurements gave 10^−3^ cm/sec [41] or 3.3 × 10^−3^ cm/sec [46]. These high estimates may have come about because the contribution of AUX importers was not appreciated. It seems likely, therefore, that auxin influx is dominated by AUX importers [44].

**Fig 2:**
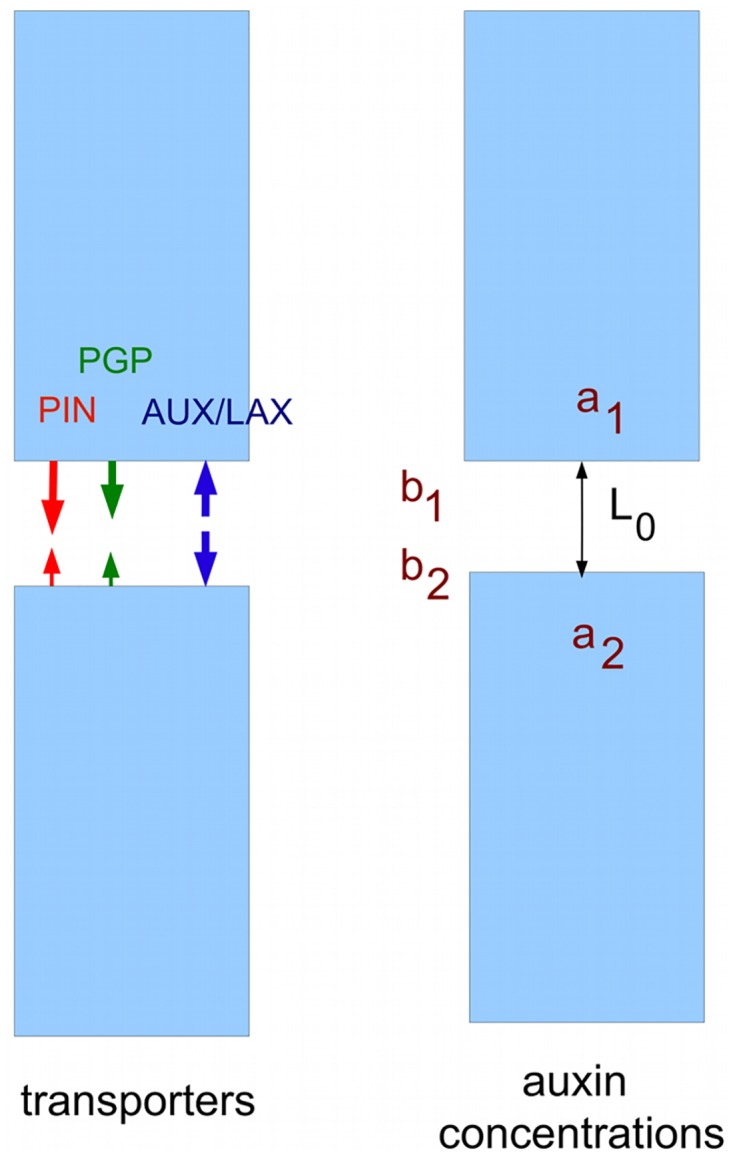
Transporters and auxin concentrations at the basal and apical ends of two cells.

Now consider two adjacent cells in a channel, and let *a*_1_ be the cytoplasmic concentrations of auxin at the basal end of the uppermost cell, and *a*_2_ the concentration at the apical end of the cell below it (see Fig 2). Let *b*_1_ and *b*_2_ be the concentrations at the apical and basal boundaries of the apoplast between the two cells, and let *L*_0_ be the vertical width of this apoplastic region. Thus we allow there to be an auxin gradient within the apoplast; let the diffusion constant of auxin in the apoplast be *D*_0_.

Let *α*_1_ be the total permeability for exporters, i.e. the sum of permeabilities for both PINs and ABCBs, in the basal cell membrane of the upper cell, and *α*_2_ the total permeability for the apical membrane of the lower cell. Let us assume that AUX/LAX importers are uniformly distributed, so the combined permeabilities for import via AUX/LAX and passive movement of protonated auxin are equal at the basal and apical membranes, and have a value *β*. The flux *ϕ* per unit area between the cells is given by

$$\phi \;=\;\alpha_{1}a_{1}-\beta b_{1},$$ (4)

$$=\;\frac{D_{0}}{L_{0}}\left(b_{1}\;-\;b_{2}\right),$$ (5)

$$=\beta b_{2}\;-\;\alpha_{2}a_{2}.$$ (6)

Eliminating *b*_1_ and *b*_2_ gives

$$\begin{array}{c} \phi =\left(\alpha_{1}a_{1}-\alpha_{2}a_{2}\right)/\left(2+r\right), \end{array}$$ (7)

where *r* = *βL*_0_/*D*_0_. This can be put in the form of Eq (1), i.e. *ϕ* = *pa*_1_ + *q*(*a*_1_ − *a*_2_), by taking

$$p\;=\;\left(\alpha_{1}\;-\;\alpha_{2}\right)/\left(2\;+\;r\right),$$ (8)

$$q\;=\;\alpha_{2}/\left(2\;+\;r\right).$$ (9)

From this we get

$$\begin{array}{c} q/p=\frac{\alpha_{2}}{\alpha_{1}-\alpha_{2}}. \end{array}$$ (10)

Since PIN1, which is probably the principal exporter in the xylem parenchyma in stems, is strongly localised at the basal end of cells [24], the contribution of PIN1 permeability to *α*_1_ will be much larger than that to *α*_2_. On the assumption that ABCBs are non-polarly distributed, the ABCB permeability contributions to *α*_1_ and *α*_2_ will be equal. However, they must surely be very small compared to the PIN1 contribution to *α*_1_, since otherwise the isotropic distribution of ABCBs implies that auxin would be pumped out at a high rate in all directions. Thus *α*_1_ must greatly exceed *α*_2_, and hence *q*/*p* must be small, highlighting the implausibility of the values of *q*/*p* in Table 1.

### Intracellular diffusion and the limitations of a single channel model

The simple model assumes complete mixing inside cells, which is unlikely to be a good approximation for the cells that transport auxin in stems and coleoptiles. Let us therefore assume that auxin diffuses through the cytoplasm with a diffusion constant D that is 60–90% [47] of its value in aqueous solution; the latter being 6.7 × 10^−6^ cm^2^/sec [48]. Suppose the cells transporting auxin have length *L*, which we take to be 100 microns. Then the analogues of Eqs (2) and (3), derived in S3 Text, are

$$\frac{1}{v}\;=\;\frac{1}{p}\;+\;\frac{L}{2D}\left(1\;+\;\frac{2q}{p}\right),$$ (11)

$$\rho \;\leq \;Lv\;(\;1\;+\;\frac{2q}{p}\;),$$ (12)

with the inequality in Eq (12) approximating an equality as *D* becomes large.

These two formulae give constraints on *q*/*p* and *D*. For instance, from the data of Morris et al. [15] we have v = 9 mm/hr and *ρ* = 10 mm^2^/hr, from which inequality Eq (12) implies *q*/*p* > 5.06, and Eq (11) gives *D* ≥ 1.4 × 10^−5^ cm^2^/sec. Thus we still have the problem of a large value of *q*/*p*, and the unrealistic requirement for instant mixing inside cells has been replaced by the requirement for a diffusion constant that is twice the measured value for diffusion in water [48]. Possibly some intracellular transport mechanism could faciliate diffusion. Cytoplasmic streaming seems not to play an important part [49], but there could be some other kind of active process, such as the movement of carriers along fibres postulated in model II in Renton et al. [22]. Against this, Kramer et al. [17] have shown that there is a broad agreement between auxin transport speeds and the bound 2*D*/*L* based on passive diffusion [16]. Moreover, even if there is enhanced diffusion, the problem of the large value of *q*/*p* still remains.

**Fig 3:**
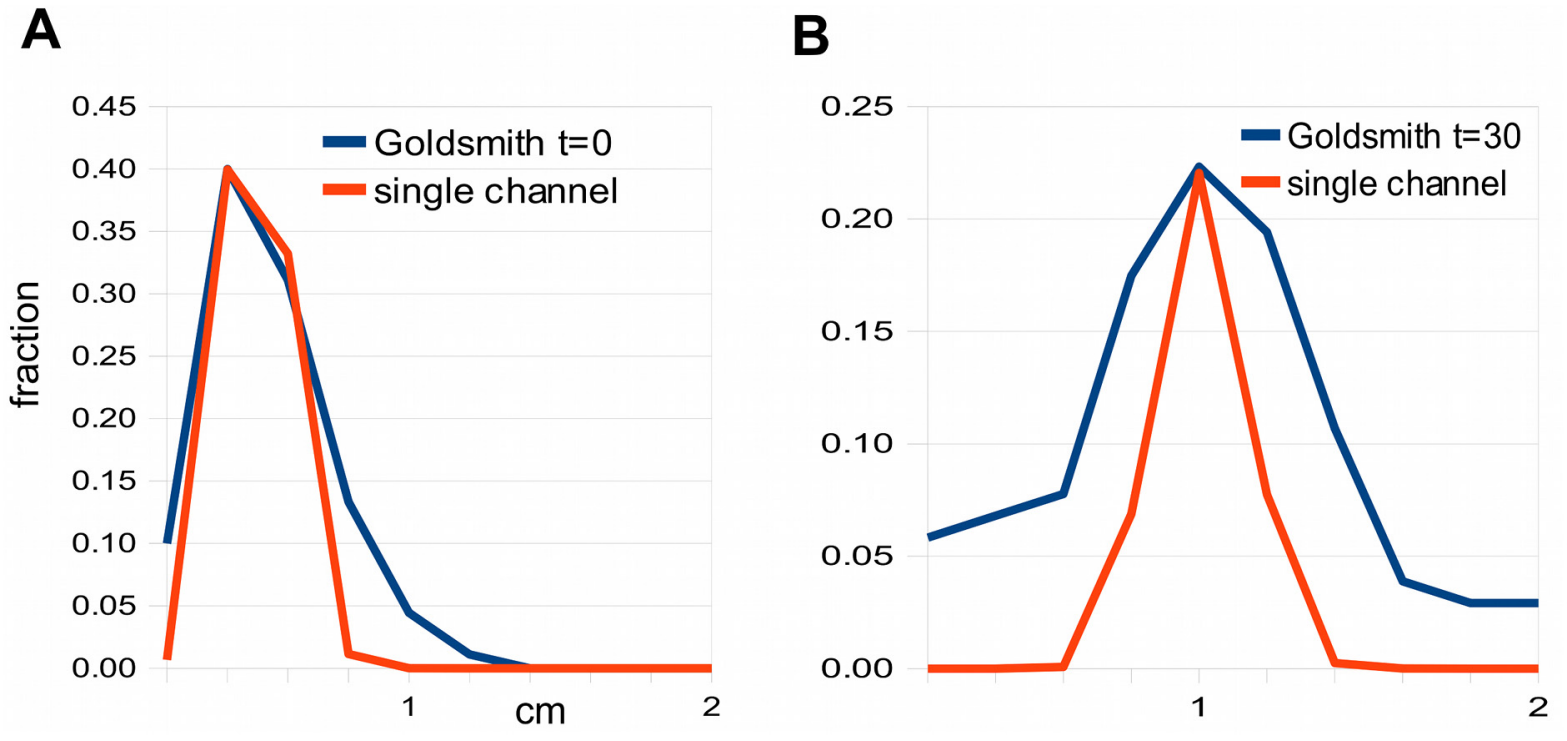
Failure of a single-channel model to account for the spreading rate in Goldsmith’s data. A: A plausible single channel model gives a reasonable fit at zero time. B: The same model gives a very poor fit at 30 mins because it cannot match the spreading rate. The model graphs have been adjusted so the peaks of model and data are the same height, to facilitate comparison of their widths. The data for panels A and B were obtained by measuring histograms in Fig 1A and 1D, respectively, in [13].

Fig 3 compares a plausible single-channel model, with *q*/*p* small and a realistic cytoplasmic diffusion constant (*q*/*p* = .05,*D* = 5 × 10^−6^ cm^2^/sec), with Goldsmith’s Fig 1A and 1D, for zero time and 30 minutes, respectively. The zero time curves match approximately, but the 30 minute single-channel distribution is much too narrow compared to the data, as expected from the arguments above.

Is there some obvious modification of the single channel model that would predict larger spreading rates? In general, any source of noise makes response curves broader, and the longer an auxin pulse runs, the more noise it would be expected to accumulate. However, incorporating various plausible sources of noise, such as random variation in cell length (so that neighbouring files are no longer in register), and randomised permeability *p*, has little effect on the spreading rate (see S4 Text).

One might also imagine that the loading of auxin from a source could have an effect on the spreading rate. Certainly, a sustained period of loading will lead to a broader pulse. However, the *rate* of broadening is unchanged, whatever the temporal pattern of loading. This is because the variance of a pulse is the sum of the variance due to an instantaneous loading event and the variance of the loading distribution (see S5 Text). The latter variance term, being a constant, disappears when we take the time-derivative to get the spreading rate.

### Saturation

Another possible explanation for a large spreading rate would be saturation of polar transporters. Saturation will cause the high concentration part of a pulse to be held back while the lower concentration part can move forwards, thus stretching out the pulse.

Consider the situation where only the PINs, usually considered to be the principal polar transporters, show saturation, all other import and export mechanisms being treated as linear. Thus the flux due to PINs obeys Michaelis-Menten kinetics of the form flux = *V*_*max*_ *a*/(*K*_*m*_ + *a*). We simplify the situation by neglecting ABCB efflux. Then instead of Eq (7) we obtain

$$\begin{array}{c} \phi =\frac{\kappa^{\left(1\right)}a_{1}}{K_{m}+a_{1}}-\frac{\kappa^{\left(2\right)}a_{2}}{K_{m}+a_{2}}, \end{array}$$ (13)

where $\kappa^{\left(i\right)}=V_{max}^{\left(i\right)}/(2+r)$, and *r* = *βL*_0_/*D*_0_.

**Fig 4:**
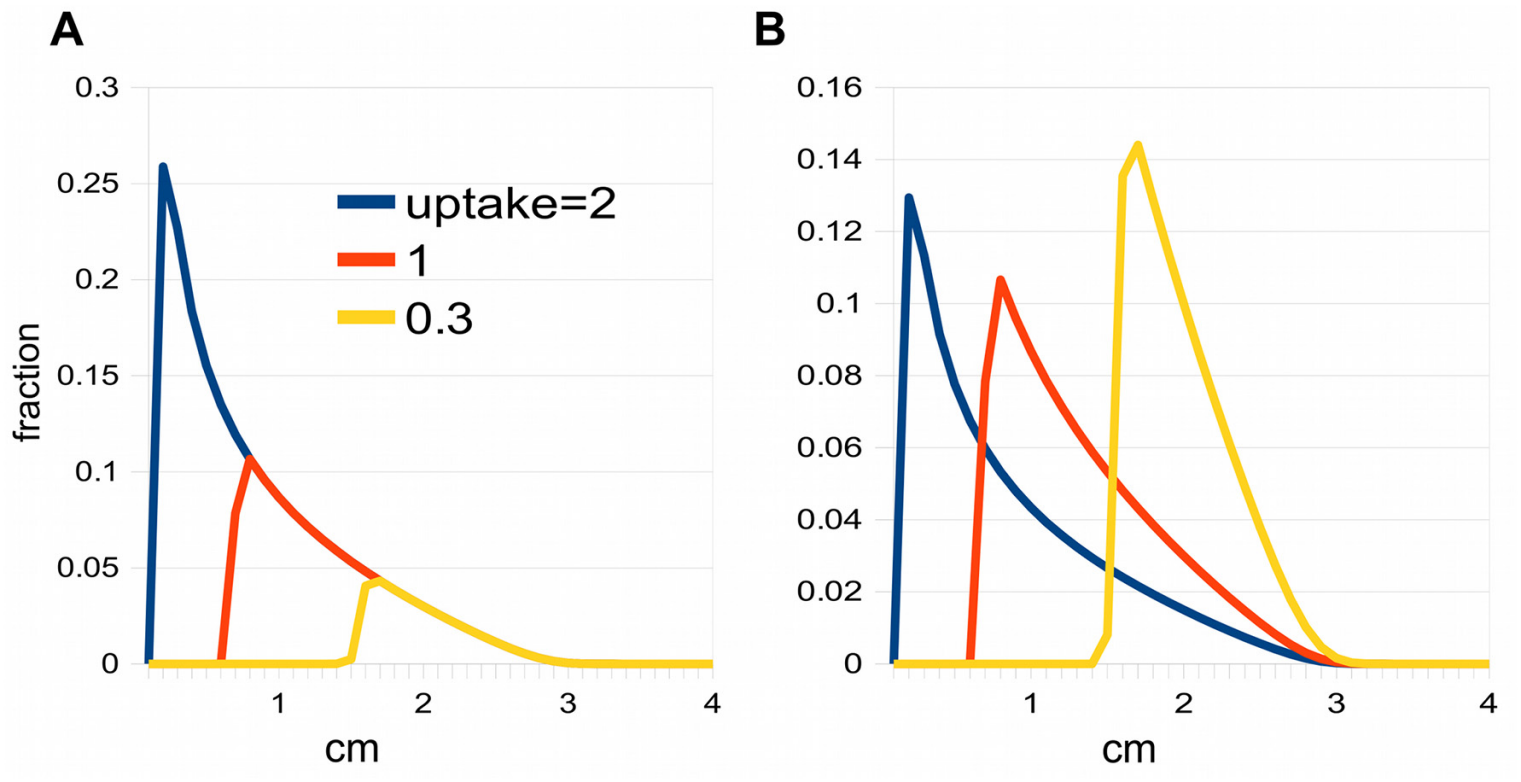
Effect of saturation on pulses. A: The three pulses have run for the same length of time. The auxin distributions have not been normalised so that the lining up of their fast fronts is apparent. The uptake is the total amount of auxin, i.e. the area under the graph. B: Same as A, but the distributions have been normalised to emphasize the peaks.

This expression for flux will come in useful later. For now, to illustrate the basic qualitative behaviour, we simplify even further by assuming that PINs are exclusively present in the basal ends of cells, so *κ* ^(2)^ = 0. This model can easily generate the required large spreading rates, by the stretching process described above. However, it leads to a characteristically-shaped pulse, with an abrupt edge at the apical end and an extended basal tail; see Fig 4. This is quite unlike the symmetrical gaussian which, as we have seen, gives a good fit to the peak region. This point is discussed in Renton et al. [22] (see their Fig 7c and 7d). In their model II, saturation of the auxin carriers can produce broad enough peaks to match the data, but the shape of the peak is then incorrect.

This suggests that saturation does not explain the spreading rate of pulses in the experiments discussed so far. However, Brewer et al. [50] showed, by using sources with differing auxin concentrations, that it was possible to change the total uptake by a factor of ten in *Arabidopsis* and almost a hundred in pea. Under these conditions, effects of saturation are seen. However, as will be shown later, these effects point to saturation of *lateral* transport, which brings us to the topic of auxin movement between multiple channels.

### Coupled channels

It appears not to be possible to generate a plausible match to the data with a single channel, and the natural next step is to consider a number of channels. If one has a collection of independent channels with a scatter of velocities, one might expect them to combine to give a pulse that grows broader as its components drift apart. However, it is unlikely that the channels are truly independent. Various experiments, such as those of Sachs [8, 9] on the induction of vascular strands by external application of auxin, suggest that auxin can move fairly freely, at least through some tissues.

One can model coupling of neighbouring cells by expressing the lateral flux per unit area formally in the same way as the axial flux, using Eq (1), or Eq (13) if there is saturation. In this section we assume that there is no saturation and also that the coupling is symmetric, so one can write

$$\begin{array}{c} \phi =s(a_{n-1}-a_{n}), \end{array}$$ (14)

where *s* is the symmetric lateral permeability and *a*_*n*−1_ and *a*_*n*_ denote auxin concentrations in cells that lie side-by-side.

**Fig 5:**
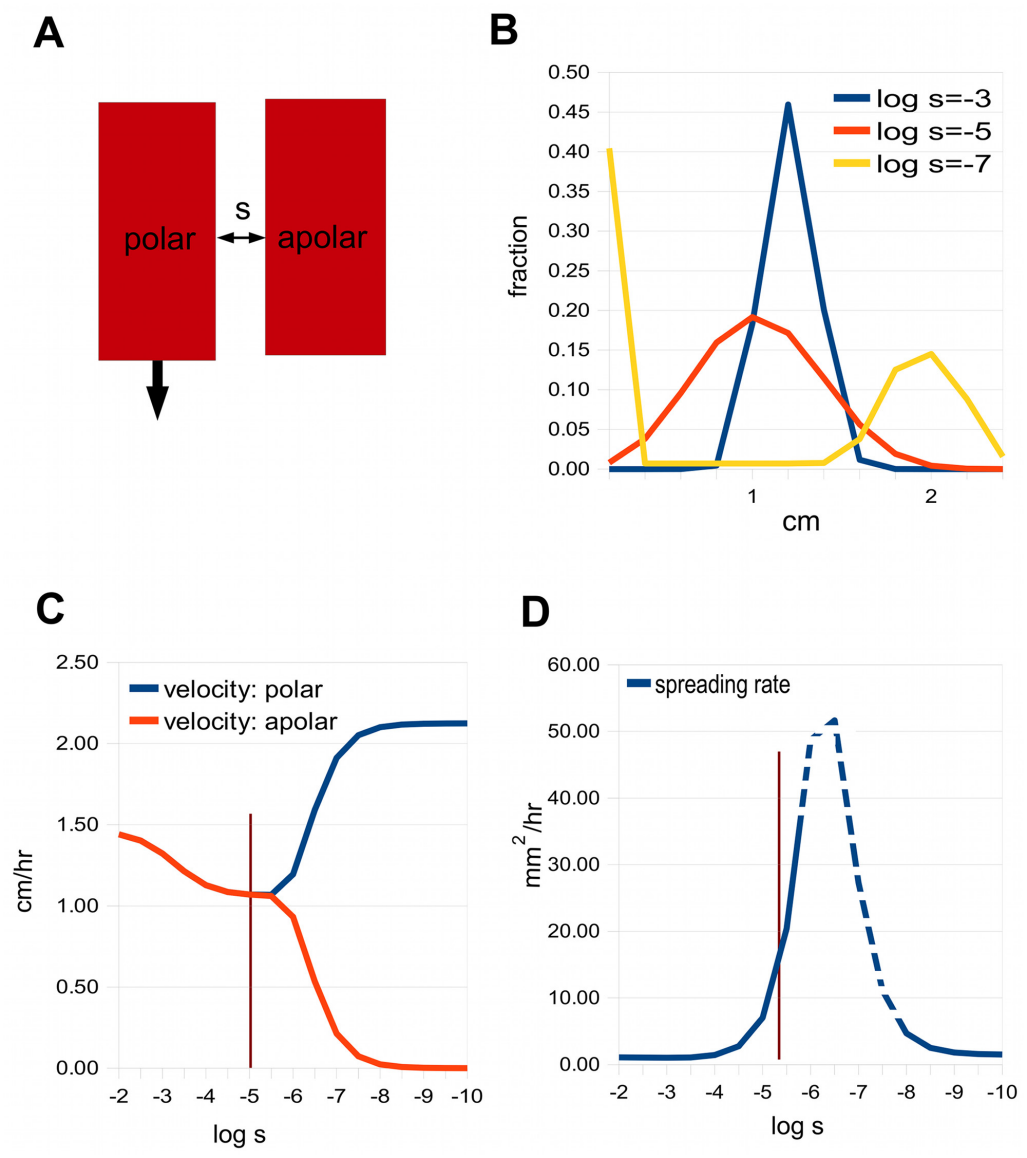
The minimal model, with two channels. A: One of the channels is polar and the other apolar, with symmetric lateral permeability *s*. B: Auxin distribution for three values of *s*, given as log_10_ *s*. C: The velocities of the two channels as a function of log_10_ *s*. The vertical line marks the value of *s* that gives the best fit to Goldsmith’s data. D: The spreading rate for the polar channel (the rates for the apolar channel are similar) as a function of log_10_ *s*. In the dotted region, the variance increases faster than linearly with time, so the spreading rate is not properly defined, and what is given is an approximation.

Consider first a minimal model that illustrates lateral coupling. It consists of two adjacent channels, one polar and the other apolar. The situation is depicted in Fig 5A. When *s* is large enough the two channels are synchronised, producing a single sharp pulse travelling at their average velocity; see the curve for log *s* = −3 in Fig 5B. As the lateral permeability decreases, the single peak eventually separates into two distinct peaks (curve for log*s* = −7). However, there is an intermediate region where the peak broadens while still keeping an essentially gaussian form (curve for log *s* = −5).

To explore this further, we observe how the velocity of the pulse in a channel varies with the lateral permeability (see Fig 5C). Starting with large *s*, the two channels have the same velocity. As *s* decreases, a critical point is reached where the velocities begin to separate, and with sufficiently small *s* the channels behave essentially separately, the apolar channel pulse having zero velocity.

Fig 5D shows the spreading rate, which rises as the lateral permeability decreases, and then eventually declines. The dotted region on the curve corresponds to a range of permeabilities where the variance does not depend linearly on time, so the spreading rate is not properly defined. This is due to a “streak” of auxin left behind the fast moving peak, which causes the variance to increase slightly faster than linearly. However, before this dotted segment is reached, the spreading rate is well-defined and can match those seen experimentally.

**Fig 6:**
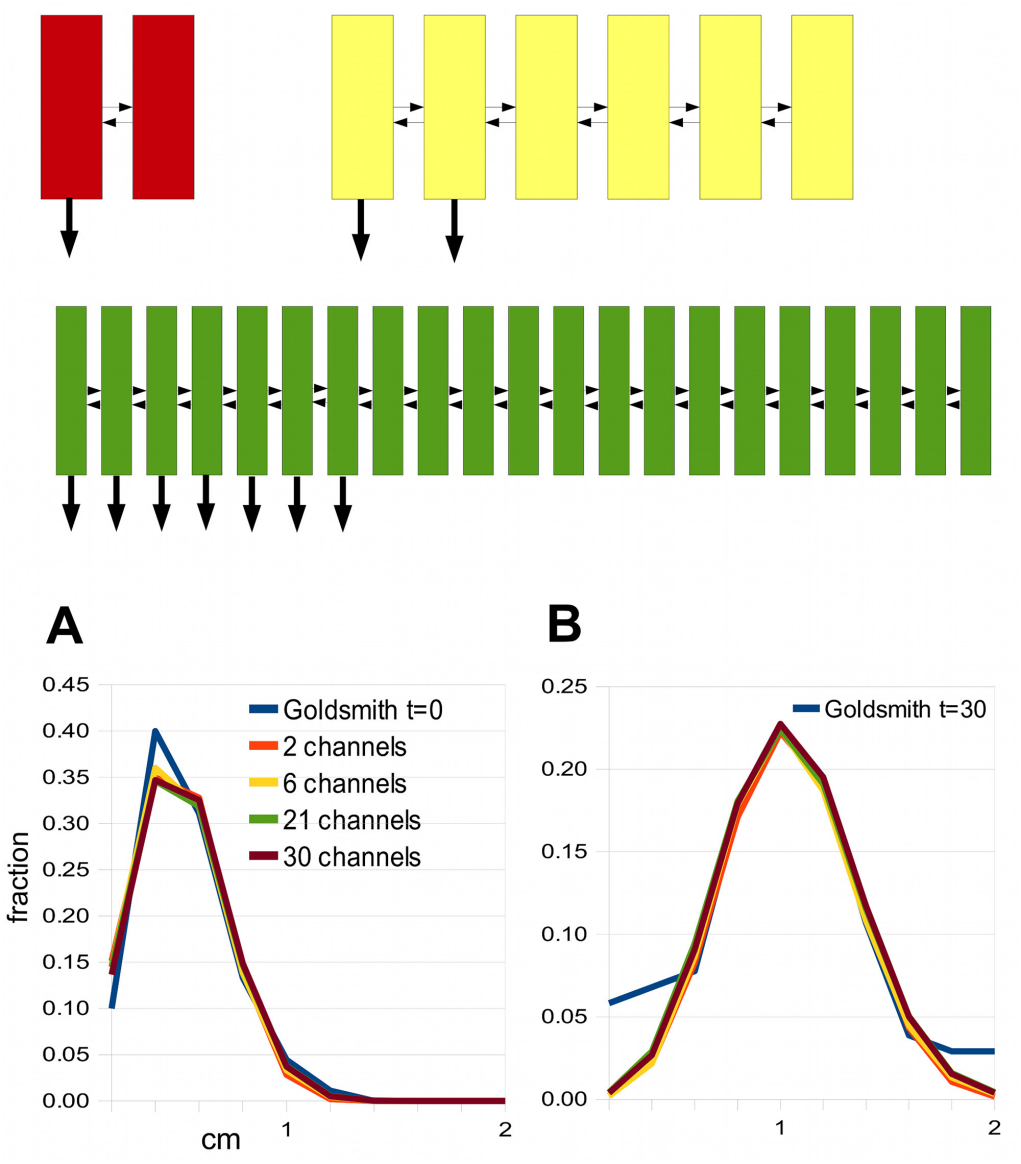
The best fits to Goldsmith’s zero-time and 30 minute pulses for the two channel minimal model and scaled-up versions of it. The thumbnail sketches show (red) the minimal model with two channels, one polar (arrow at the basal end), the other apolar; (yellow) a model with 2 polar and 4 apolar channels; (green) a model with 7 polar and 14 apolar channels. In all cases, the small horizontal arrows indicate the lateral coupling. A: Zero-time pulse from each model (and also a model with 30 channels). B: 30 minute pulse from each model. They are compared with data obtained from Fig 1A and 1D in [13].

We use Goldsmith’s data as a test set for various models. The vertical line in Fig 5C and 5D marks the value *s* = 7.1 × 10^−6^ cm/sec, where the 2-channel model gives a reasonable fit (by eye) to her Fig 1A and 1D at zero time and 30 minutes, respectively. Fig 6A and 6B show the corresponding auxin distributions.

One can scale this two-channel model up to larger numbers of channels, where there is a group of polar channels adjacent to a group of apolar channels (see the thumbnail sketches in Fig 6). This might be the situation, for example, when there is a specialised transporting tissue adjacent to a tissue where auxin moves diffusively. In these scaled-up models, the lateral coupling is assumed to be symmetric and of equal strength, *s*, everywhere. Thus one can regard *ws*, where *w* is the width of a cell, as a diffusion coefficient for lateral movement. More precisely, diffusion within cells with rate *D* will combine with the intercellular coupling [51] so the effective diffusion constant, *D*_*eff*_, is given by:

$$\begin{array}{c} \frac{1}{D_{eff}}=\frac{1}{D}+\frac{1}{ws}. \end{array}$$ (15)

Now one intuitive explanation for the broadening of a pulse by coupling of channels is that it is due to the lateral diffusion of auxin. The diffusion distance, or the average distance that a molecule diffuses in time *t*, is $2\sqrt{Dt}$, (e.g. expression (3.40) in [52]). If one is comparing models with varying numbers of channels, and hence varying total width *L*, the time needed to diffuse the distance *L* is given by $L=2\sqrt{Dt}$, or $t=\frac{L^{2}}{4D}$. So if the amount of broadening depends on this time *t*, we expect models to give similar shapes of pulses when the diffusion constant, *D*_*eff*_, is proportional to *L* ^2^, or the square of the number of the channels. One can test this by finding the best matches to our test set for various numbers of channels; see Fig 6. The resulting values of *D*_*eff*_ do indeed scale in a quadratic manner, and the same is true for *s* when *ws* ≪ *D*, since the term 1/(*ws*) then dominates 1/*D* in Eq (15). However, the scaling only holds approximately (Table 2). It seems this intuition only partially captures the complicated dynamics.

**Table 2:** Scaling of the lateral permeability needed to match Goldsmith’s data with the number of channels.

n	Total width	*s* cm/sec	*D*_*eff*_ cm^2^/sec	quadratic scaling
2	40 *μ*	7.1 × 10^−6^	1.4 × 10^−8^	1.4 × 10^−8^
6	120 *μ*	8.0 × 10^−5^	1.6 × 10^−7^	1.3 × 10^−7^
21	0.42 mm	1.4 × 10^−3^	1.8 × 10^−6^	1.5 × 10^−6^
30	0.6 mm	1.2 × 10^−2^	4.1 × 10^−6^	3.2 × 10^−6^

The lateral permeability, *s*, and the corresponding effective diffusion constant needed to match Goldsmith’s data, for various values of *n*, the number of channels. The models are sketched in Fig 6. The last column shows the values of *D*_*eff*_ expected from scaling by *n* ^2^. For the first three values of *s*, i.e. for *n* = 2,6,21, the term 1/(*ws*) dominates 1/*D* in Eq (15), and one sees approximate quadratic scaling of *s*.

Note that there is a limit to lateral movement set by diffusion within the cytoplasm. From an engineering point of view, to achieve rapid lateral movement without wasted resources, the permeability *s* for movement between cells should be matched to diffusion within them. This means that the equivalent diffusion constant, *ws*, (where *w* is the width of a cell) should be equal to the diffusion constant *D* for auxin in cytoplasm, or equivalently that the two terms in Eq (15) should be equal. Taking *w* = 20 microns and *D* = 5 × 10^−6^ cm^2^/sec, this gives *s* = 2.5 × 10^−3^ cm/sec, which is not far from the best-fitting value for the 21-channel model (Table 2). With this value, *D*_*eff*_ = 2.5 × 10^−6^ cm^2^/sec, which means that auxin can move rapidly enough through tissues to register changes at distances of the order of a millimetre within an hour (since *t* = *L* ^2^/*D*_*eff*_ with L = 1 mm is 1.1 hrs), which seems biologically reasonable. The biological parallel for this engineering argument would be that evolutionary pressure to achieve rapid signalling would lead to increasing values of the permeability *s*. However, at the point where *ws* = *D*, increasing *s* gives ever smaller gains in *D*_*eff*_, and selection for larger values of *s* becomes weak.

**Fig 7:**
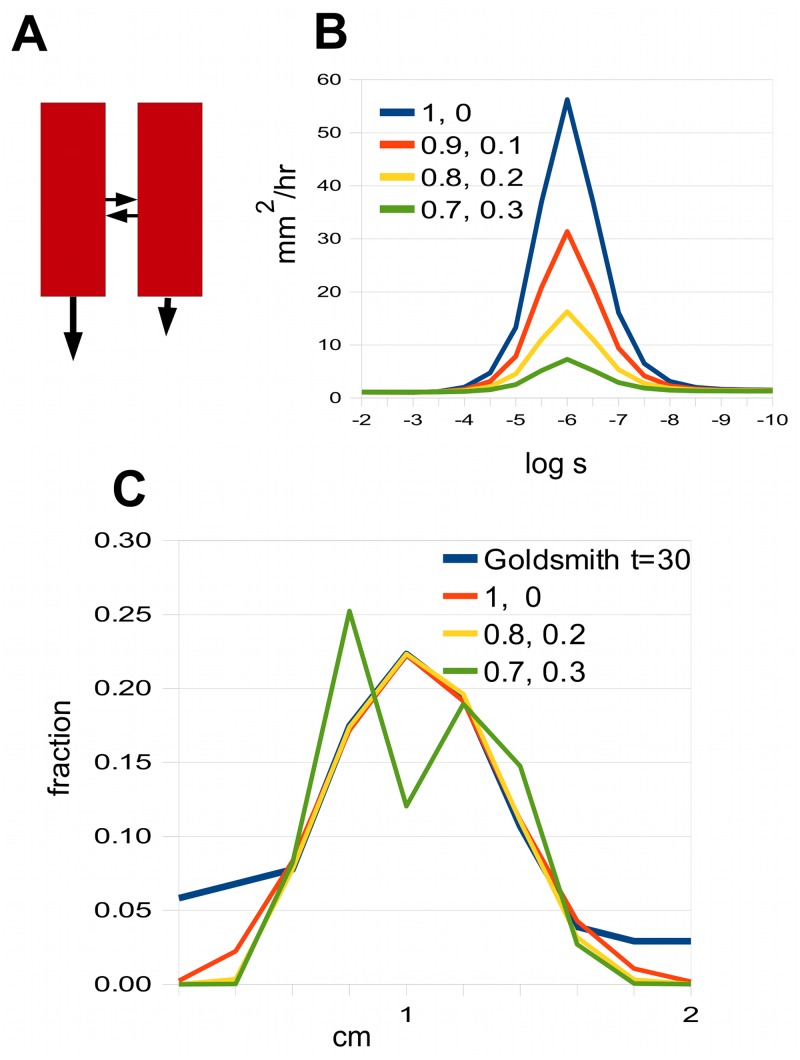
Spreading rates for modified version of the minimal model. A: The two channels are both polar but with different degrees of polarity (different sized vertical arrows). B: The spreading rate for some values of the ratio of the polarities of the channels. C: Best fits to Goldsmith’s 30 minute data for different polarity ratios, showing that how it fails when the polarity ratio falls to 0.7:0.3. The curve labelled “Goldsmith” is based on data in Fig 1D in [13].

There are many other ways of varying the minimal model that still allow a good fit to Goldsmith’s data. For instance, one can consider two channels that are both polar, but with strengths that are in some ratio *α* : 1−*α* (Fig 7A). One can plot the spreading rate against coupling strength (as in Fig 5D), and Fig 7B shows a number of such graphs for different values of the ratio *α* : 1−*α*. As can be seen, the maximum spreading rate decreases as the polarities of the two channels become more similar. This implies that one can only find a match to Goldsmith’s data when the polarities are sufficiently different. Fig 7C shows that there is a match for 0.8:0.2, but not for 0.7:0.3, where the peak splits as the coupling is weakened in an attempt to get a sufficiently large spreading rate.

### Coupling between intracellular compartments

**Fig 8:**
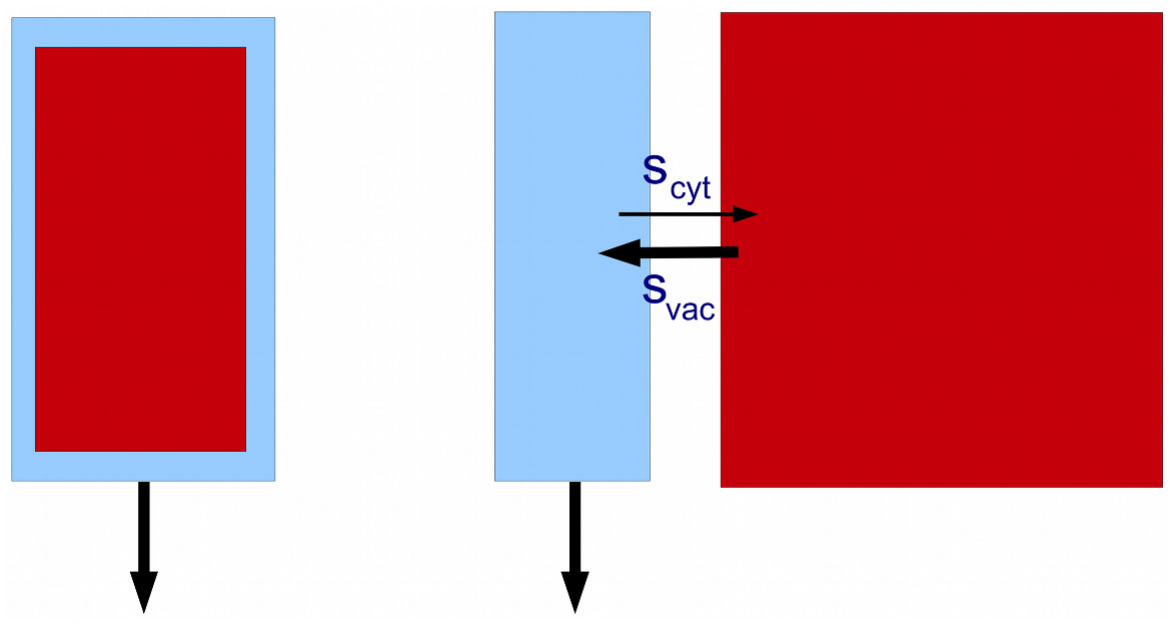
Treating the vacuole and cytoplasm as channels. The vacuole (red) can be regarded as an apolar channel coupled to the cytosol (blue).

The spreading of a pulse due to lateral movement through the cells of a tissue could in principle also be produced by local movement *within* a cell: instead of looking at coupling between cells, we can look at coupling between subcellular compartments. One obvious example is the vacuole, which takes up most of the volume of a plant cell, in mature tissues at least. We can represent the cytosol/vacuole pair by the minimal model, i.e. Fig 5A, with *n* = 2. The cytosol is the polar channel and the vacuole the apolar channel; they are coupled laterally via the tonoplast (Fig 8). One difference is that there is no coupling between successive cells in the vacuolar channel, i.e. *p* = *q* = 0 for apical and basal faces, whereas the apolar channel in the minimal model has the same permeability on all cell faces (See Models, Fig 6). However, setting the apical and basal permeabilities to zero has only a very small effect on the dynamics, especially for the case *n* = 2 where *s* is small (Table 2).

Another example is the endoplasmic reticulum (ER). Auxin is thought to be moved from the cytosol to the ER by PIN5 [27], and PIN6/PIN8 may move auxin in the reverse direction [28]. Again we can represent the cytosol/ER pair by the minimal model, with PINs 5/6/8 providing the lateral coupling. The question is whether the minimal model, applied to these compartments, can account for the observed spreading rate, or for some fraction of it (lateral movement between and within cells could both be operating at the same time).

Consider first the vacuole. Two new features have to be added to the minimal model: asymmetry in the size of the compartments, and also a possible asymmetry in the inward and outward permeabilities, so in place of Eq (14), or *ϕ* = *s*(*a*_1_−*a*_2_) for the flux *ϕ* from channel 1 (cytosol) to channel 2 (vacuole), we have *ϕ* = *s*_*cyt*_ *a*_1_−*s*_*vac*_ *a*_2_, where *s*_*cyt*_ is the permeability from cytosol to vacuole, and *s*_*vac*_ is the permeability in the opposite direction. In the minimal model it was assumed that each channel (cell file) was 20 microns wide. Let us assume that the cytosol and vacuole have widths *d*_*cyt*_ and *d*_*vac*_ microns, respectively. We can rescale the permeabilities to take account of these widths, so that the dynamics of the best-fitting minimal model will be reproduced if we take *s*_*cyt*_ = *s*(*d*_*cyt*_/20), *s*_*vac*_ = *s*(*d*_*vac*_/20), where *s* is given by Table 2, *n* = 2, as 7.1 × 10^−6^ cm/sec.

Let us pause to consider the geometry of the cell and its vacuole. A cell is represented computationally by a brick shape whose long axis measures 100 microns and the two sides 20 microns; thus it has a square cross-section. The cytoplasm is assumed to form a thin layer, 1 micron in depth, just inside the boundary of the cell. Thus the vacuole to cytoplasm volume ratio is 18^2^ × 98:20^2^ × 100−18^2^ × 98, or about 4:1. This is low compared to the average of about 10 : 1 [53], but probably more typical for the elongated xylem parenchyma cells that transport auxin efficiently in the stem. In comparing *d*_*cyt*_ and *d*_*vac*_, we need to take into account that there are two layers of cytoplasm on opposite sides of the cell, so we can pair each layer of cytoplasm with the adjacent half of the vacuole, whose width is half that of the whole vacuole, namely 9 microns. Thus we take *d*_*cyt*_ = 1 and *d*_*vac*_ = 9. With these values, and with the best-matching *s* we get *s*_*cyt*_ = *s*(*d*_*cyt*_/20) = 3.6 × 10^−7^ cm/sec and *s*_*vac*_ = *s*(9*d*_*vac*_/20) = 3.2 × 10^−6^ cm/sec.

Thus we obtain a good match to Goldsmith’s data if *s*_*cyt*_ and *s*_*vac*_ have the above values. We can compare them with the values expected from passive diffusion. With a pH for the cytoplasm of 7.2 [43]), and using *p*_*IAAH*_ = 4 × 10^−5^ cm/sec [45], we get *s*_*cyt*_ = 1.2 × 10^−7^ cm/sec. Similarly, taking the pH of the vacuole to be 5.5 [42, 43, 54], we find *s*_*vac*_ = 5.6 × 10^−6^ cm/sec. As can be seen, these are of the same order as the best-fitting values. However, *s*_*cyt*_ is about 3 times smaller and *s*_*vac*_ almost twice as large as these best-fitting values, so there is a bias towards excluding auxin from the vacuole.

These calculations show that, given passive diffusion, only about a quarter of the auxin in a cell resides in the vacuole, despite its large relative volume. The effect of this exclusion is that coupling with the vacuole produces only a small amount of broadening of the pulse: about 2.6 mm^2^/hour with passive diffusion. In addition to passive diffusion there may also be auxin transporters in the tonoplast; indeed, there is evidence for an exporter from the vacuole, the WAT1 transporter [55] (though see also [56]). It seems possible that a combination of low pH and exporters ensures that auxin is largely excluded from the vacuole, in which case its contribution to the spreading rate will of course be negligible.

**Fig 9:**
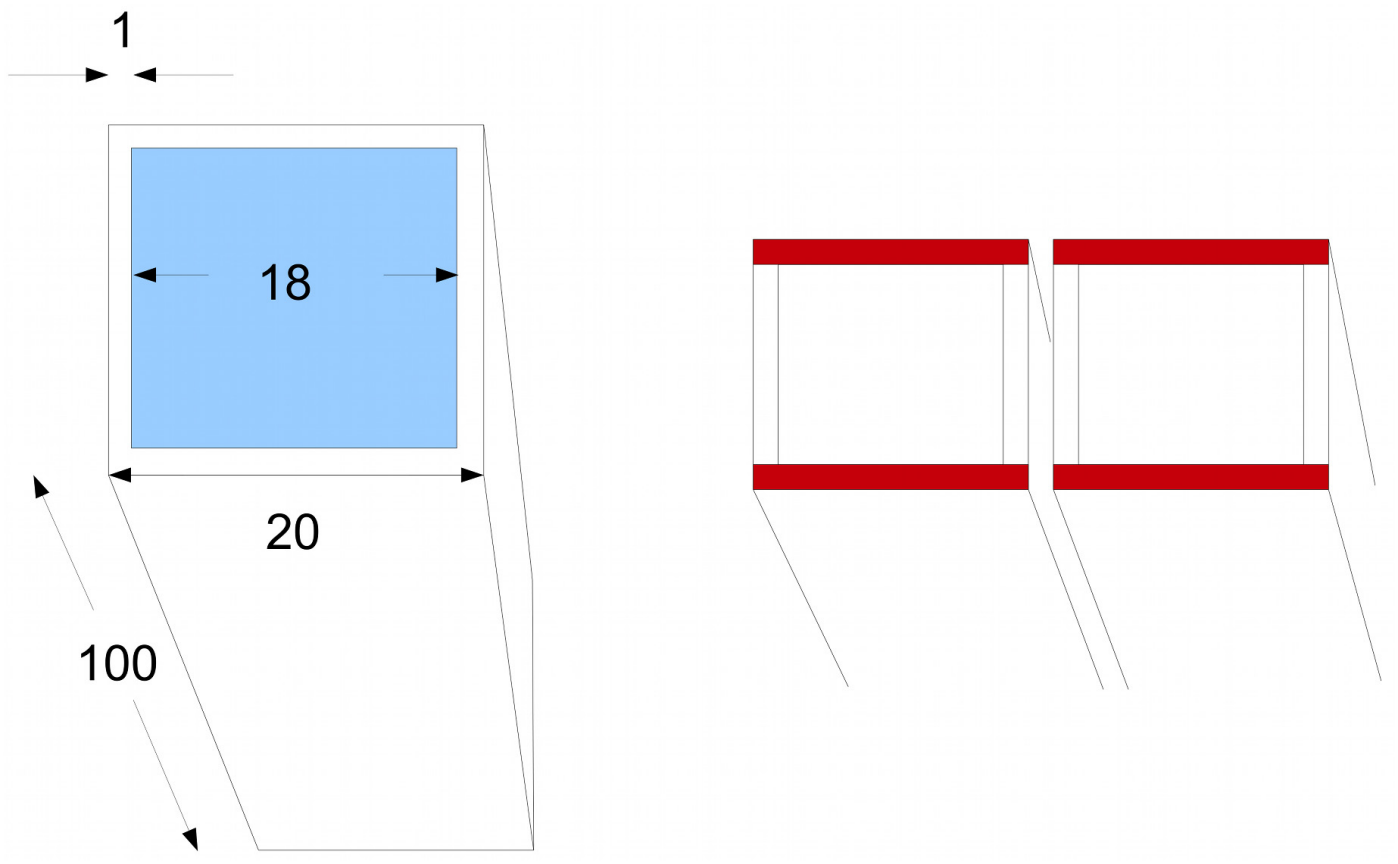
Geometry of the vacuole. Left: a cell represented for computational purposes as a brick shape with a square cross section. The vacuole is in blue. Measurements in microns. Right: we consider lateral coupling in the horizontal direction. The cytoplasmic layers in red can be regarded as the routes for lateral flux.

We take a final look at the geometry of the vacuole to explain why it is plausible, when looking at lateral movement between cells, to treat each cell as having a 20 micron width, despite the vacuole filling much of the space. The reason is that one can view lateral flux as taking place in sheets of cytoplasm 1 micron thick but 20 microns wide within each cell, and with corresponding sheets in adjacent cells (marked in red in Fig 9). The cytoplasmic sheets at right angles serve as an extended interface between adjacent cells. This is of course a gross oversimplication, but at least provides a tractable computational model.

Turning next to the ER, if auxin is both imported and exported by specific channels [28], then the permeabilities are likely to be much larger than for passive diffusion, so we will be in the left-hand part of the graph in Fig 5D where the spreading rate is low. Thus if we regard the ER simply as a space through which auxin diffuses, it is unlikely to explain the spreading rate. However, there is evidence that auxin undergoes metabolic processing once it enters the ER [27], and this could plausibly account for the frequently observed accumulation of label in the wake of a pulse (see for instance Fig 8 in [14]).

### Saturation revisited

**Fig 10:**
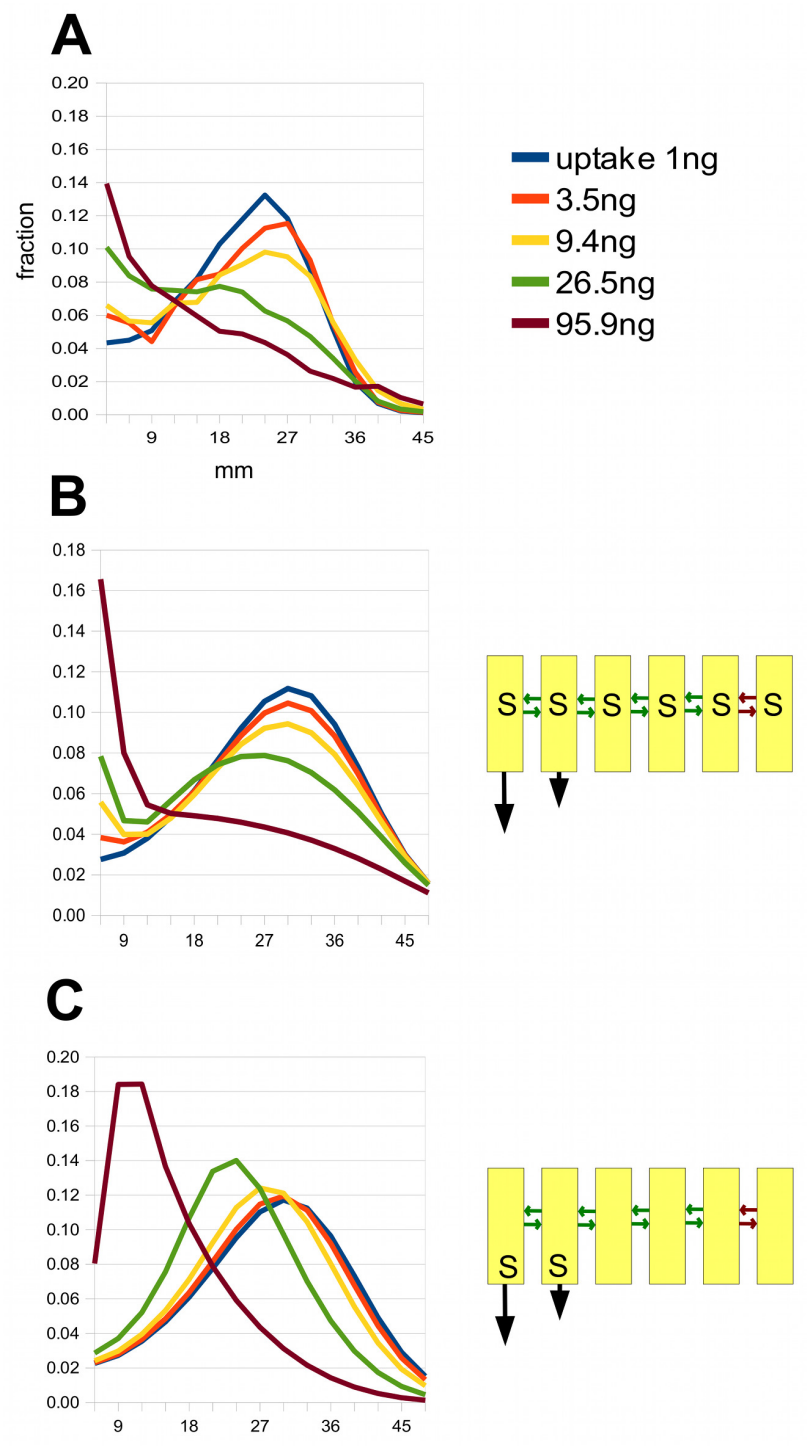
Modelling the apical build-up of auxin as the uptake increases. A: This shows auxin distributions in the pea for various total uptakes after 4 hours, and is based on data from Fig 4A–4E in Brewer et al. [50]. The uptakes in nanograms come from summing the counts in the original data (communicated by Dr Ferguson). All distributions are normalised to 1. B: A simulation of these data using the 6-channel model shown in Fig 6 and in the sketch on the right, but introducing saturation in the lateral transporters, indicated by an ‘S’ in the cells. The green arrows indicate weak diffusive coupling between channels, and the red arrows an even weaker coupling between the 5th and 6th channels. C: A version of the model in B with the same parameters but with saturation in the axial polar transporters and not in the lateral transporters.

We now return to the question of saturation. The pea data of Fig 4A–4E in Brewer et al. [50], kindly supplied by Dr Brett Ferguson (S1 Data), are redrawn in Fig 10A. They show how the shape of a pulse changes when increasing amounts of auxin are applied and larger amounts of auxin taken up by plant tissues (determined by the total counts in the stem segments). There is a gradual build-up of auxin near to the apical end of the stem which gradually swamps the peak as the uptake increases.

This does not resemble the effect of saturation of the polar transporters in a single channel model described earlier, where the trailing edge of the pulse remains sharp as the uptake increases (Fig 4). A better match to the experimental data can be obtained by assuming that there is saturation of the *lateral* movement between channels. As a first try at modelling this, S6 Text, we add lateral saturation to the n = 6 version of the model shown in Fig 5 (with the minor modification that we make the two polar channels of different strengths, which gives a better eventual fit). We assume that auxin initially moves from the externally applied source into the apolar channels, from which it can move laterally into the polar channels. The idea is that, as the uptake of auxin increases, lateral transport will be limited by saturation, and a diffusion gradient of residual auxin in the apolar channels will be created, accounting for the apical build-up seen in the data. There is no saturation of the axial (polar) transporters, only of those that move auxin laterally.

S6A Fig shows how this model behaves. The 26.5 ng peak is retarded relative to the peaks with smaller loadings. There is also a marked apical build-up with the 95.9 ng pulse. However, there is little or no apical build-up with the lower uptakes, as there is in the pea data. Weakening the lateral coupling partially brings this about but distorts the curves in other ways. However, selectively weakening of the lateral coupling between the fifth and sixth channels, as shown in Fig 10B, does achieve a fairly good fit to the data. Thus a model of modest complexity can capture the qualitative features of the family of data curves.

If we take this model and keep all parameters unchanged except that we switch the saturation from lateral to axial transporters, the simulated pulses are as shown in Fig 10C. This is clearly a very poor fit to the data, with large shifts in the positions of peaks, like those seen in Fig 4, and no build-up at the apical end.

## Discussion

Finding an explanation for the large spreading rate of auxin pulses turns out to be an intriguing puzzle. One can rule out a single-channel model, and also variants of it that introduce noise, e.g. by randomising cell length or the degree of polarity in successive cells in the channel. These models fail by a large margin to achieve a large enough spreading rate.

I propose here that the explanation lies in the coupling together of several, perhaps many, channels of different degrees of polarity. When the coupling strength is large, the channels are essentially bound together into one “super-channel” that has too sharp a peak to match the data. When the coupling is sufficiently weak, pulses in the different channels move at different velocities and separate. There is, however, an intermediate range of lateral coupling strength where the pulses in all the channels move coherently but the resulting pulse spreads out.

It is this intermediate coupling regime that matches the data best. A good fit can only be obtained if the coupled channels have sufficiently disparate polarities. This might mean, for example, that some of them correspond to specialised polar transport tissue and others to apolar, or very weakly polar, tissues. Alternatively, the specialised transport tissues themselves might consist of channels with varying degrees of polarity, due to their stage of maturation for example. There is also a wide range of possible physical scales that can produce the observed phenomena, ranging from a minimal model of two channels (with a total width of 40 microns) to extended models with 20–30 parallel channels (0.4 to 0.6 mm).

This brings home the fact that there are many ways of modelling the same data, which is hardly surprising, given that one is only fitting two parameters: the velocity and spreading rate of a pulse. There are more constraints once one brings saturation into the picture and is required to explain not just the peak but also the build-up at the apical end when large amounts of auxin are taken up, as with the data from Brewer et al.; Fig 10A). The six-channel model of Fig 6 can be adapted to give a good match to the data, by introducing saturation of lateral permeabilities and weak coupling between some of the channels, which allows them to act as a slowly emptying reservoir of auxin that is waiting to enter the rapid transport stream (Fig 10B).

However, even with this more complex scenario, the model is not unique. An alternative model, S6 Text and S6B Fig, can achieve as good a match. In this model there is an asymmetry in the lateral permeabilities, which ensures that auxin can only enter, and not exit from, the polar channels. There is some evidence for this kind of polar lateral transport (e.g. [57], Fig 1), and it could in principle have a strong influence on the lateral distribution of auxin. Thus the difference between the two models bears on the problem of balancing basipetal flow with lateral propagation.

Although the channels have been envisaged as files of cells, it is also possible that they could be compartments within cells. However, it has been argued that two conspicuous compartments, the vacuole and the endoplasmic reticulum, are unlikely to play this role, the former because the coupling with the cytosol is too weak, the latter for the opposite reason. Thus the cell file interpretation seems more plausible, and one attractive conjecture is that the 21-channel expanded version of the minimal model, with a corresponding physical width of 0.4 mm, comes closest to reality for the coleoptile, as it fits not only Goldsmith’s data but also accords with the ‘engineering’ criterion of matching lateral diffusion between channels to diffusion within cells.

An intriguing final observation is that the best-fitting parameters for Goldsmith’s data imply a coupling strength that is close to the point where the velocities in the channels begin to separate; the vertical line in Fig 5C lies close the split. A similar conclusion applies to the data of Morris et al. [15]. This suggests that the polar transport system is only just able to carry along with it the auxin flow in the surrounding apolar tissues. This could be an indication of energetic economy, or perhaps a way of creating responsiveness to changes.

## Models

In all the models, cells are assumed to be 100 microns long and 20 microns wide. To simulate intracellular diffusion, cells are represented as rectangles divided up lengthwise into *N* compartments, where N was generally taken to be 5. If lateral coupling constants are large enough so that lateral intracellular diffusion needs to be taken into account, this is done by a further lengthwise subdivision of each compartment into two.

A permeability having a computational value of *p*′ is converted into a permeability p measured in cm/sec by

$$\begin{array}{c} p=p^{\prime }\frac{L}{N\Delta }, \end{array}$$

where L is the cell length in cm and Δ is the time step, generally taken to be 1/20 sec. The computational parameter *D*′ for intracellular diffusion is converted into a diffusion constant D in cm^2^/sec by

$$\begin{array}{c} D=D^{\prime }\frac{L^{2}}{N(N-1)\Delta }. \end{array}$$

This makes Eq (11) equivalent to

$$\begin{array}{c} \frac{1}{v^{\prime }}=\frac{1}{p^{\prime }}+\frac{N-1}{2D^{\prime }}(1+\frac{2q^{\prime }}{p^{\prime }}), \end{array}$$

as observed in [16] (see the passage following Eq (40)).

The constants and simulation details for the models used for generating the figures are as follows:

Fig 3. For the single channel model in this figure, *p* = 4 × 10^−4^ cm/sec, *q* = 2.08 × 10^−5^ cm/sec, *D* = 5 × 10^−6^cm^2^/sec.

Fig 4A Here we take $V_{max}^{\left(i\right)}=10^{-6}$ cm^2^/sec, *K*_*m*_ = 10^−3^, *q* = 0, and *D* = 5 × 10^−6^ cm^2^/sec. The total amount of auxin uptake, treated as an instantaneous pulse, takes the values 0.3, 1 and 2 in arbitrary units. The simulation runs for the equivalent of 90 minutes. B The same data as in A normalised.

Fig 5 For the polar channel, *p* = 1.4 × 10^−3^cm/sec, *q* = 0; for the apolar channel *p* = *q* = 0. For all channels, *D* = 5 × 10^−6^ cm^2^/sec. It could be argued that *q* should be comparable to *s*, since an apolar cell should have uniformly distributed permeabilities. However, we wish to study the effect of changing the lateral coupling alone, so *q* is fixed at zero. And in fact, the picture does not change qualitatively if we put *q* = *s* rather than *q* = 0. Uptake of auxin was simulated by adding at each computational step a fixed amount to the concentration in all the channels at the apical end for the loading period. At the end of the loading period, the distribution was normalised to a total of 1. We used Goldsmith’s protocol for loading, with 15 minutes of uptake at a constant rate at the apical end of the segment. This was followed by 45 minutes of transport, corresponding to Goldsmith’s 30 minute data, since she includes a 15 minute wait after loading before “zero time”. In the dotted region of the graph of spreading rate in D, a trail of auxin behind the frontal peak makes the variance increase faster than linearly, so the spreading rate is not defined. Instead, what is plotted is (Var(0)-Var(t))/t, where Var(t) denotes the variance of the distribution at time t, and t is taken to be 30 mins. If one had chosen t to be 15 mins, for example, the solid-line parts of the curve would have been identical, and the dotted line part slightly lower.

Fig 6 For all polar channels, *q* = 0. For apolar channels we take *p* = 0 and put *q* = *s*, where *s* is the value of the lateral permeability needed to match Goldsmith’s data given the number of channels in the model. Thus permeabilities are the same on all cell faces in apolar cells. For the polar channels we take the following values of *p*: *n* = 2, *p* = 1.4 × 10^−3^ cm/sec, *n* = 6, *p* = 6 × 10^−3^ cm/sec, *n* = 21 *p* = 6.4 × 10^−3^cm/sec, and *n* = 30, *p* = 8.8 × 10^−3^cm/sec. In all cells *D* = 5 × 10^−6^ cm^2^/sec. Loading follows the protocol given above for Fig 5. Graphs in B were scaled so that the peak heights coincide.

Fig 7 To make the graph on the left, the two-channel model is used with *p* = 1.44 × 10^−3^, so *p*_*left*_ = *αp*, and *p*_*right*_ = (1−*α*)*p*, where *α*:1−*α* takes the values 1 : 0, 0.9 : 0.1, etc., indicated on the graph. For both channels, *q* = 0, *D* = 5 × 10^−6^ cm^2^/sec. To make the right-hand graph, we again take *q* = 0, *D* = 5 × 10^−6^ cm^2^/sec in both channels, and now seek values of *p* and *s* that optimise the fit (or come as close as possible), for the indicated ratios *α*:1−*α*. For 1:0 we take *p* = 1.44 × 10^−3^ cm/sec, *s* = 1.4 × 10^−5^ cm/sec. For 0.8:0.2 we take *p* = 9.6 × 10^−4^ cm/sec, *s* = 9.6 × 10^−6^ cm/sec. For 0.7:0.3 we take *p* = 8.8 × 10^−4^ cm/sec, *s* = 4.0 × 10^−7^ cm/sec.

Fig 10B Saturation kinetics is used only in the lateral walls (marked by an ‘S’ in the sketch). The lateral coupling between the channels is symmetric, with *κ*_1_ = *κ*_2_ = *K*_*m*_ *s* in Eq (13), where *K*_*m*_ = 10^−4^ and *s* = 2.4 × 10^−5^ cm/sec for the green arrows and 4 × 10^−7^ cm/sec for the red arrows. The other (unsaturated) permeabilities are *p* = 3.2 × 10^−3^ cm/sec in channel 1, *p* = 4 × 10^−4^ cm/sec in channel 2, and for channels 3 to 6 *p* = 0 and *q* = 8 × 10^−3^ cm/sec. For all channels, *D* = 5 × 10^−6^cm^2^/sec. Loading is assumed to be instantaneous in the initial computational cycle and to be into channels 2 to 6 only. The computation runs for the equivalent of 4 hours. The parameters in C are as in B, but now saturation kinetics are used for the axial permeability, so in Eq (13) for channels 1 and 2 we have *κ*_1_ = *K*_*m*_ *p*, *κ*_2_ = 0. There is no saturation in the lateral coupling.

## Supporting Information

S1 Data Data underlying Fig 4E and 4F (WT pea only) in Brewer et al. [50], Fig 1A–1D and 1G–1H from Goldsmith [13], and individual plant data contributing to the averaged curves shown in Fig 4 of Morris et al. [15]. (XLS)

S1 Text Selecting the peak region from noisy data. Using least-squares and the maximal-fit algorithm to find the peak region in various data sets.
(PDF)

S2 Text The velocity and spreading rate for the simple model. Calculations of the expressions for *v* in Eq (2) and *ρ* in Eq (3).
(PDF)

S3 Text The velocity and spreading rate with intracellular diffusion. Calculations of the expressions for *v* in Eq (11) and *ρ* in Eq (12).
(PDF)

S4 Text The effect of noise on the spreading rate. Variability in model parameters has little effect on spreading rate.
(PDF)

S5 Text Loading does not affect the velocity or spreading rate. The velocity and spreading rate are unchanged by any time-varying auxin uptake process.
(PDF)

S6 Text Modelling apical build-up of auxin. Further exploration of models for the data of Brewer et al. [50].
(PDF)

S1 Fig Least-squares fitting of a gaussian to a pulse. This shows a gaussian fitted by least-squares to a 30 minute auxin distribution from Goldsmith’s pulse experiments. The y-axis shows the proportion of total auxin present in each 2 mm segment. Based on Goldsmith’s data from Fig 1D in [13].
(TIF)

S2 Fig Fitting noisy data. Fig 4 in Morris et al. [15] shows pulses that are the average from a number of segments. Here a pulse from an individual segment is shown (data kindly supplied by Dr Morris), revealing more noise than in the averaged data. A: Least squares compared with selection by eye. B: maximal fit (S1 Text) compared with selection by eye, showing much better agreement.
(TIF)

S3 Fig Further examples of successful maximal fit. Reasonable concordance between the peak fitted by eye and the maximal fit algorithm (S1 Text): individual segment data for 5.2 hours underlying the averaged graph in Fig 4 in Morris et al. [15].
(TIF)

S4 Fig Maximal fit performs less well at short times. Less good agreement between eye and maximal fit for 2 hour individual segment data from [15].
(TIF)

S5 Fig The function *f* which gives a correction to the spreading-rate. The function *f*(*pL*/*D*,*q*/*p*), with *pL*/*D* on the x-axis, and for various values of *q*/*p*; see S3 Text. For the simulations, the number of intracellular compartments was taken to be *N* = 10 (see Models). A pulse was initiated in a single compartment and allowed to progress down the stem for 30 minutes, its variance measured, and then, after a further 30 minutes of transport, measured again, thus allowing *ρ* to be estimated accurately. The function *f* was then calculated from Eq (S8).
(TIF)

S6 Fig Modelling the apical build-up with increasing auxin uptake. See S6 Text. A: First attempt at modelling the data in Fig 4A-4E in Brewer et al. [50] using the 6-channel model of Fig 6, with equal lateral permeability between all channels, i.e. without a weaker coupling between channels 5 and 6 as in Fig 10. B: A four-channel model that also produces a good fit to the data. Note the absence of an arrow from channel 2 to channel 3. In this model, the (unsaturated) permeabilities are *p* = 8 × 10^−4^ cm/sec in channel 1, *p* = 10^−4^ cm/sec in channel 2, and for channels 3 to 6 *p* = 0 and *q* = 2 × 10^−3^ cm/sec. The lateral, saturable, permeabilities have *K*_*m*_ = 10^−4^, and *s* = 2 × 10^−6^ cm/sec for the green arrows and 5 × 10^−7^ cm/sec for the red arrows. For all channels, *D* = 3 × 10^−6^cm^2^/sec.
(TIF)
