# Supplementary material for: The Shape of an Auxin Pulse, and What It Tells Us about the Transport Mechanism
Source: PLoS Comput Biol. 2015 Oct 20;11(10):e1004487. doi: 10.1371/journal.pcbi.1004487 (PMC4618354; doi:10.1371/journal.pcbi.1004487)
Supplement: S1 Text — Using least-squares and the maximal-fit algorithm to find the peak region in various data sets. (PDF) [file pcbi.1004487.s002.pdf]

Selecting the peak region from noisy data

S1 Fig shows that least-squares fitting can effectively pick out the peak region of a pulse in Goldsmith's data [1]. Formally, least-squares means we minimize

$$S = \sum_i (a_i - g_i)^2,$$

where a_i is the measured auxin concentration, i.e. the counts in a piece of the stem, and

$$g_i = \frac{1}{\sqrt{(2\pi s)}} e^{-(i-m)^2/(2st)}$$

is a gaussian with mean m and variance s sampled at the integral point i. When the auxin profile gets more complex, as in the single segment data underlying Fig 4 in [2], this method performs less well; see S2A Fig. The fitted gaussian tries to encompass more points than the peak region itself, which was chosen by eye as the region marked in red.

An alternative that works better is to maximize

$$T = \sum_i a_i e^{-\alpha(a_i - g_i)^2}, \tag{S1}$$

where α is some constant. Here, mismatches are not directly penalized; a good score is achieved by having a close fit in some region where the auxin concentration a_i is high, i.e. near to the peak. This means that the gaussian is able to follow the peak and neglect outlying values.

One flaw with this method is that the fitted gaussian can have values greater than the counts over an extended region without being penalised. There is an asymmetry in our requirements: we don't mind the gaussian being smaller than the data values; we do mind it being larger. This is because the gaussian is meant to represent a subset of counts that are attributed to the main auxin channel. To capture this asymmetry, we use the following modification of Eq (S1):

$$S = \sum_{g_i \le a_i} a_i e^{-\alpha(a_i - g_i)^2} - \beta \sum_{g_i > a_i} (g_i - a_i), \tag{S2}$$

where α and β are suitable constants. We call this the *maximal fit* algorithm, the 'maximal' implying that we choose the gaussian by maximising S.

This method is undoubtedly ad hoc. One can also pick the peak region by eye, independently of any algorithm. S3 Fig and S2B Fig show all the data sets for 5 hours, with a good match between the region chosen by eye and maximal fit.

The match is less good for the 2 hour data sets, S4 Fig, where the problem of identifying the peak is greater. However, the estimated velocities and spreading rates do not differ much for peaks picked by eye or by maximal fit.

References

1. Goldsmith MHM. Movement of Pulses of Labeled Auxin in Corn Coleoptiles. Plant Physiol. 1967;42:258-263.

2. Morris SE, Cox MCH, Ross JJ, Krisantini S, Beveridge CA. Auxin dynamics after decapitation are not correlated with the initial growth of axillary buds. Plant Physiol. 2005;138:1665-1672.
