# Supplementary material for: The Shape of an Auxin Pulse, and What It Tells Us about the Transport Mechanism
Source: PLoS Comput Biol. 2015 Oct 20;11(10):e1004487. doi: 10.1371/journal.pcbi.1004487 (PMC4618354; doi:10.1371/journal.pcbi.1004487)
Supplement: S2 Text — Calculations of the expressions for v in Eq (2) and ρ in Eq (3). (PDF) [file pcbi.1004487.s003.pdf]

The velocity and spreading rate for the simple model

Recall that, in the simple model, flux is given by $\phi = pa_{n-1} + q(a_{n-1} - a_n)$. Assuming there is no creation or destruction of auxin within cells, this implies

$$L\frac{da_n}{dt} = p(a_{n-1} - a_n) + q(a_{n-1} + a_{n+1} - 2a_n),$$

where L is the length of a cell. If the distribution describes a pulse, then the distance, μ, that its mean travels in time t is given by

$$\mu = \sum_n nLa_n / \sum_n a_n \tag{S1}$$

and the velocity, v, is the derivative of the mean:

$$\begin{aligned}
v = d\mu/dt &= \left(\sum nLda_n/dt\right) / \sum a_n \\
&= \sum n\Big[p(a_{n-1} - a_n) + q(a_{n-1} + a_{n+1} - 2a_n)\Big] / \sum a_n, \\
&= p\Big[\sum(n-1)a_{n-1} + \sum a_{n-1} - \sum na_n\Big] / \sum a_n \\
&\quad + q\Big[\sum(n-1)a_{n-1} + \sum a_{n-1} + \sum(n+1)a_{n+1} - \sum a_{n+1} - 2n\sum a_n\Big] / \sum a_n, \\
&= p\sum a_n / \sum a_n, \\
&= p,
\end{aligned} \tag{S2}$$

which is Eq (2).

To compute the spreading rate, ρ, recall that this is defined to be $\rho = d(Var)/dt$, where the variance is given by:

$$Var = \frac{\sum_n (nL - \mu)^2 a_n}{\sum_n a_n} = \frac{\sum_n n^2L^2a_n}{\sum_n a_n} - \mu^2. \tag{S3}$$

We have

$$\begin{aligned}
d/dt\sum n^2L^2a_n &= \sum n^2L^2da_n/dt \\
&= L\sum n^2\left(p(a_{n-1} - a_n) + q(a_{n-1} + a_{n+1} - 2a_n)\right) \\
&= L(p+q)\sum[(n-1)^2 + 2(n-1) + 1]a_{n-1} - L(p+2q)\sum n^2a_n \\
&\quad + Lq\sum[(n+1)^2 - 2(n+1) + 1]a_{n+1}, \\
&= 2Lp\sum na_n + L(p+2q)\sum a_n,
\end{aligned}$$

where the last step follows if we ignore end effects, so

$$\begin{aligned}
\sum(n-1)a_{n-1} &= \sum(n+1)a_{n+1} = \sum na_n \\
\sum(n-1)^2a_{n-1} &= \sum(n+1)^2a_{n+1} = \sum n^2a_n.
\end{aligned}$$

Thus

$$\begin{aligned}
\rho = \frac{d(Var)}{dt} &= \frac{\sum n^2L^2da_n/dt}{\sum a_n} - 2\mu\frac{d\mu}{dt}, \\
&= 2p\mu + L(p+2q) - 2p\mu, \\
&= L(p+2q),
\end{aligned} \tag{S4}$$

which is Eq (3).

One can also write down a continuous version of the model:

$$\frac{\partial a}{\partial t} = -p\frac{\partial a}{\partial x} + q\frac{\partial^2 a}{\partial^2 x}, \tag{S5}$$

which has a gaussian solution

$$a = \frac{1}{\sqrt{2\pi qt}} e^{-(x-pt)^2/2qt}. \tag{S6}$$

This explains why a gaussian is such a good approximation in the discrete case.
