# Supplementary material for: The Shape of an Auxin Pulse, and What It Tells Us about the Transport Mechanism
Source: PLoS Comput Biol. 2015 Oct 20;11(10):e1004487. doi: 10.1371/journal.pcbi.1004487 (PMC4618354; doi:10.1371/journal.pcbi.1004487)
Supplement: S3 Text — Calculations of the expressions for v in Eq (11) and ρ in Eq (12). (PDF) [file pcbi.1004487.s004.pdf]

The velocity and spreading rate with intracellular diffusion

We would like to express the velocity, v, of an auxin pulse in terms of the permeabilities p, q for the movement of auxin between cells, and the diffusion constant, D, for movement of auxin inside a cell. This can be done surprisingly simply. Imagine a continuous source at one end of a segment with a receiver removing auxin at the other end. We can think of the equilibrium distribution as a sum of many small pulses, each due to the uptake from the source for a time Δt. They will overlap to produce a constant distribution, with an identical linear concentration gradient inside each cell. Suppose the concentration at the apical end of each cell is a and at the basal end is b. Then there are three expressions for ϕ, the flux per unit area of auxin down the segment:

$$\phi = \frac{D(a-b)}{L}, \tag{S1}$$

$$= pb + q(b-a), \tag{S2}$$

$$= \frac{v(a+b)}{2}. \tag{S3}$$

The first, Eq (S1), gives the flux due to diffusion inside the cell; the second, Eq (S2), expresses the flux in terms of transport between two cells; the third, Eq (S3), gives the flux in terms of the average velocity, v, of the underlying pulses, multiplied by the average concentration inside a cell. Eliminating a, b and ϕ from these three equations gives

$$\frac{1}{v} = \frac{1}{p} + \frac{L}{2D}\left(1 + \frac{2q}{p}\right), \tag{S4}$$

which is Eq (11). The formula was first derived in [1]. Note that it makes the assumption that there is a constant velocity for a pulse. This is not the case when end-effects and initial conditions are taken into account, as shown mathematically in Martin et al. [2]. However, the velocity obtained from computations is always in good agreement with the formula, so the assumption of constant velocity seems sound when modelling experiments.

Now consider the spreading rate, ρ in the presence of intracellular diffusion. We can regard it as being due to two components, spreading within cells and spreading due to coupling of cells ignoring the intracellular diffusion, i.e. with infinitely rapid mixing. The former is given by the gaussian solution of the diffusion equation, namely

$$f(x,t) = \frac{K}{t^{1/2}} e^{-x^2/(4Dt)},$$

which has variance $2Dt$ and hence spreading rate $2D$. The latter is given by Eq (3) as $L(p+2q)$. It is tempting to think that the spreading rate of the composite model should be the sum of these component spreading rates. However, this is incorrect, as one sees in the case where D becomes large. Instead, one should consider the inverses of the spreading rates, which can be regarded as the rate of change of the elapsed time with respect to variance. Because of interactions between the two components this only gives a lower bound for $1/\rho$:

$$\frac{1}{\rho} \geq \frac{1}{2D} + \frac{1}{L(p+2q)}. \tag{S5}$$

This can be rewritten as

$$\rho \leq \frac{L(1+2q/p)}{\frac{1}{p} + \frac{L(1+2q/p)}{2D}}, \tag{S6}$$

or, using Eq (S4),

$$\rho \leq vL(1 + 2q/p), \tag{S7}$$

which is Eq (12).

This is a bound, but not an exact formula for ρ. In the absence of an analytical expression, one can compute the ratio:

$$f(pL/D, q/p) = \frac{\rho}{vL(1 + 2q/p)}, \tag{S8}$$

which depends only on the two dimensionless variables pL/D and q/p. From Eq (S7), $f \leq 1$. However, computer exploration shows there is also a lower bound, $f \geq 0.67$. The function f is close to 1 when $pL \ll D$ and when q/p is large. Its behaviour can be seen in S5 Fig, which was generated by running a single channel model with a range of values of the two parameters pL/D and q/p.

There is another way to derive Eq (S7), which is to assume that, during the propagation of a pulse, diffusive equilibrium is reached inside cells, so that one can apply Fick's law, Eq (S1), for the flux, but to allow the mean concentration within cells to vary with position according to the shape of the pulse. As shown by Kramer [3] (his Eqs A4 and A5), the flux can then be written as a sum of two components, one due to the velocity of the pulse and the other due to an effective diffusion constant, D_{eff}, for movement of auxin between adjacent cells, where $D_{eff} = \frac{1}{2}vL(1+2q/p)$. In other words, D_{eff} is half the bound on the righthand side of Eq (S7). If one assumes that the auxin distribution follows the gaussian solution for a pulse spreading under diffusion, where the variance of the gaussian is $2Dt$ (see [4], Eq (2.6)), then one expects $\rho = 2D_{eff}$, which gives the correct formula. However, it is not clear from this argument that $2D_{eff}$ is an upper bound, only that it is an approximation that is valid when diffusive equilibrium can be assumed inside cells, which corresponds to small values of pL/D.

References

1. Mitchison GJ (1980) The dynamics of auxin transport. Proc. Roy. Soc. Lond. B 209:489-511.
2. Martin MH, Goldsmith MHM, Goldsmith TH (1990) On polar auxin transport in plant cells. J. Math. Biol. 28:197-223.
3. Kramer EM (2002) A mathematical model of pattern formation in the vascular cambium of trees. J. Theor. Biol. 216:147-158.
4. Crank J (1975) The mathematics of diffusion. The Clarendon Press. Oxford.
