# Supplementary material for: The Shape of an Auxin Pulse, and What It Tells Us about the Transport Mechanism
Source: PLoS Comput Biol. 2015 Oct 20;11(10):e1004487. doi: 10.1371/journal.pcbi.1004487 (PMC4618354; doi:10.1371/journal.pcbi.1004487)
Supplement: S4 Text — Variability in model parameters has little effect on spreading rate. (PDF) [file pcbi.1004487.s005.pdf]

The effect of noise on the spreading rate.

One can introduce randomness into the model and ask how it affects spreading rate. For instance, one can replace a fixed value of p by random selection from a uniform distribution from the interval $p/2$ to $3p/2$ (which maintains the same average value of p). Taking the best fitting 2-channel model illustrated in Fig 6, where the spreading rate is 9.60 mm^2/hr, randomizing p in this fashion gives a distribution of spreading rates with mean 8.98 and standard error of the mean (SEM) from 100 trials of 0.05. Thus the spreading rate is, with high probability, *reduced* by this source of noise. Doing the same with s increases the spreading rate to 9.74 with SEM 0.13. Finally, one can allow the cell length to vary randomly. Instead of taking $L = 100$ microns, we choose L with equal probability from 60, 80, 100, 120, and 140 microns. This means in particular that the two cell files in the model are no longer in register. Again the spreading rate *decreases*, with mean 8.72 with SEM 0.07.

Thus there are only small effects on the spreading rate as a result of quite substantial variability in model parameters. Moreover, these effects are not always even positive.
