# Supplementary material for: The Shape of an Auxin Pulse, and What It Tells Us about the Transport Mechanism
Source: PLoS Comput Biol. 2015 Oct 20;11(10):e1004487. doi: 10.1371/journal.pcbi.1004487 (PMC4618354; doi:10.1371/journal.pcbi.1004487)
Supplement: S5 Text — The velocity and spreading rate are unchanged by any time-varying auxin uptake process. (PDF) [file pcbi.1004487.s006.pdf]

Loading does not affect the velocity or spreading rate

Loading can be described by a function $\phi(t)$ which gives the rate of uptake of auxin from the source at time t. We assume that loading is complete after some time τ, so $\phi(t) = 0$ for $t > \tau$, and that the loading function is normalised, so that $\int_0^\tau \phi(t)dt = 1$.

We show that the velocity and spreading rate measured after time τ are the same as for instantaneous loading. Suppose the the pulse produced after time t following instantaneous loading is $\psi(x,t)$. We assume the pulse is normalised, in the sense that $\int_{-\infty}^{\infty} \psi(x,t)dx = 1$. We assume the instantaneously loaded pulse function travels at velocity v, so

$$\int_{-\infty}^{\infty} x\psi(x,t)dx = vt, \tag{S1}$$

and has spreading rate ρ, so

$$\int_{-\infty}^{\infty} (x - vt)^2\psi(x,t)dx = \rho t. \tag{S2}$$

The total pulse at time T after loading with the function $\phi(t)$ is

$$f(x,T) = \int_0^T \psi(x, T - t)\phi(t)dt.$$

Using Eq (S1), the mean of the pulse at time $T > \tau$ is

$$m = \int xf(x,T)dx = v\int_0^T (T - t)\phi(t)dt = v(T - \bar{t}),$$

where $\bar{t}$ is the mean of the loading function. Thus the velocity of the pulse is $dm/dT = v$, which is independent of the loading function.

The variance is

$$\begin{aligned}
\text{var} &= \int_{x=-\infty}^{\infty} (x - vT + v\bar{t})^2 f(x,T)dx, \\
&= \int_{x=-\infty}^{\infty}\int_0^T (x - vT + v\bar{t})^2\psi(x, T - t)\phi(t)dtdx, \\
&= \int_{x=-\infty}^{\infty}\int_0^T \left[(x - vT + vt)^2 + 2v(\bar{t} - t)(x - vT) + v^2(\bar{t}^2 - t^2)\right]\psi(x, T - t)\phi(t)dtdx, \\
&= (T - \bar{t})\rho + v^2\int_0^T (t - \bar{t})^2\phi(t)dt.
\end{aligned}$$

The integral in the second term is the variance of the loading function if $T > \tau$. Thus the only term depending on T is $T\rho$, and therefore the spreading rate dvar/dT is ρ. This is independent of the loading function.
