# Supplementary material for: The Shape of an Auxin Pulse, and What It Tells Us about the Transport Mechanism
Source: PLoS Comput Biol. 2015 Oct 20;11(10):e1004487. doi: 10.1371/journal.pcbi.1004487 (PMC4618354; doi:10.1371/journal.pcbi.1004487)
Supplement: S6 Text — Further exploration of models for the data of Brewer et al. [50]. (PDF) [file pcbi.1004487.s007.pdf]

Modelling apical build-up of auxin.

The simplest way of adapting the 6-channel model (Fig 6) to model the data of Brewer et al. [1] is to introduce weak, saturable coupling between the channels, as shown in S6A Fig. The retarding of the 26.5 ng pulse seen in this model can be interpreted as follows: As the uptake increases, and hence the intracellular auxin concentrations rise, saturation makes the lateral permeability effectively smaller. Our previous analysis of the effect of changing coupling strength, Fig 5C and D, tells us that this should decrease the velocity and increase the spreading rate. Both these effects can be seen with the 26.5 ng pulse compared to the lower uptakes. This is consistent with the pea data. However, other features of the pea data, such as the apical build-up of the 26.5 ng apical pulse, are not captured by this model.

A more complete match to the data can be achieved by decreasing the strength of the coupling between the two channels furthest from the polar channels (see Fig. 10B); the most distant channel then acts as a reservoir of auxin that only enters the transport stream slowly. However, it is striking that a good match to the data can also be obtained from a rather different model, which has only four channels and features a one-way lateral permeability into the polar channels (S6B Fig).

References

1. Brewer PB, Dun EA, Ferguson BJ, Rameau C, Beveridge CA. Strigolactone acts downstream of auxin to regulate bud outgrowth in pea and *Arabidopsis*. Plant Physiol. 2009;150:482-493.
