# Supplementary figures and images for: The Shape of an Auxin Pulse, and What It Tells Us about the Transport Mechanism
Source: PLoS Comput Biol. 2015 Oct 20;11(10):e1004487. doi: 10.1371/journal.pcbi.1004487 (PMC4618354; doi:10.1371/journal.pcbi.1004487)

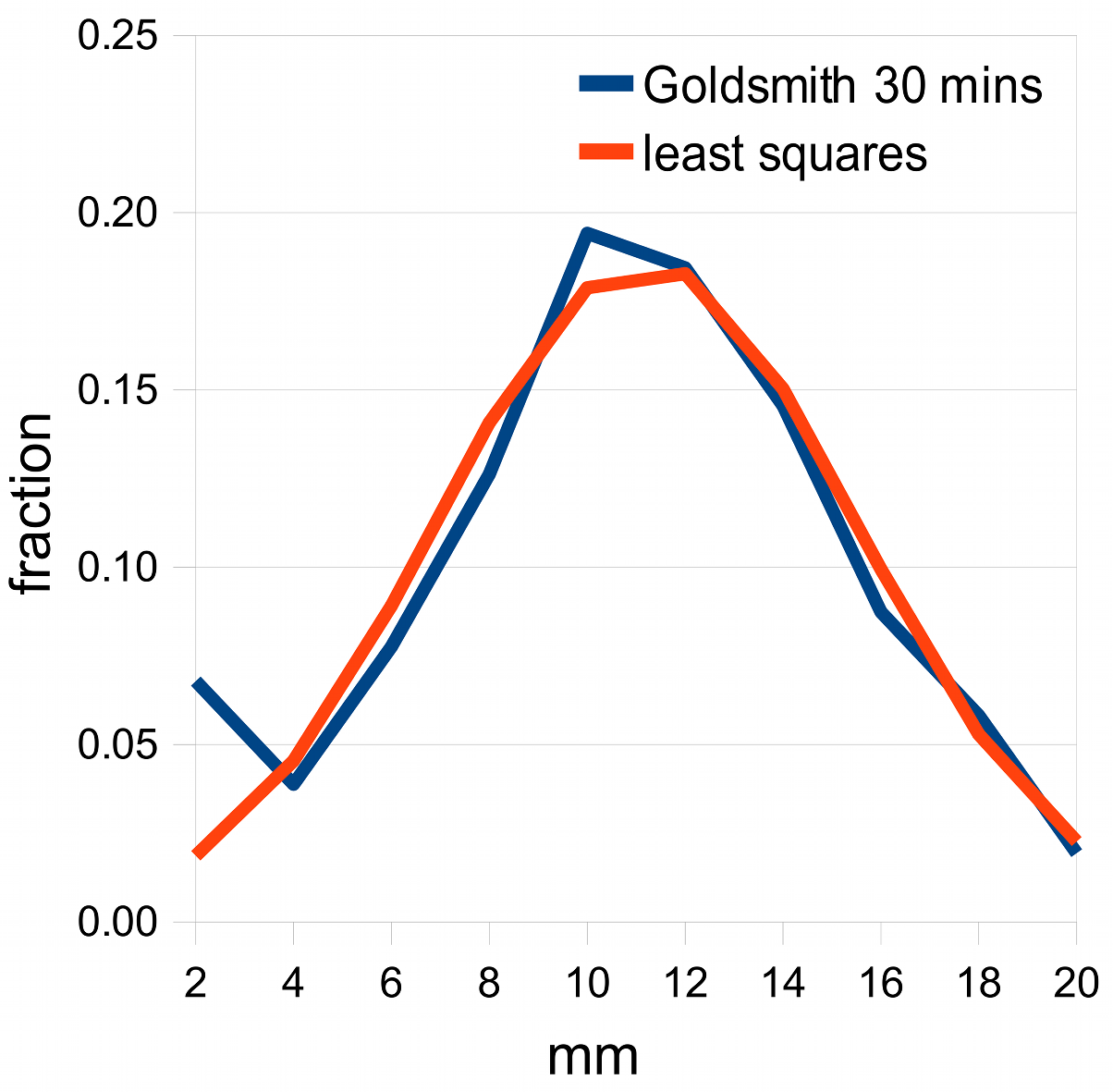

Supplement: S1 Fig — This shows a gaussian fitted by least-squares to a 30 minute auxin distribution from Goldsmith’s pulse experiments. The y-axis shows the proportion of total auxin present in each 2 mm segment. Based on Goldsmith’s data from Fig 1D in [13]. (TIF) [file pcbi.1004487.s008.tif]

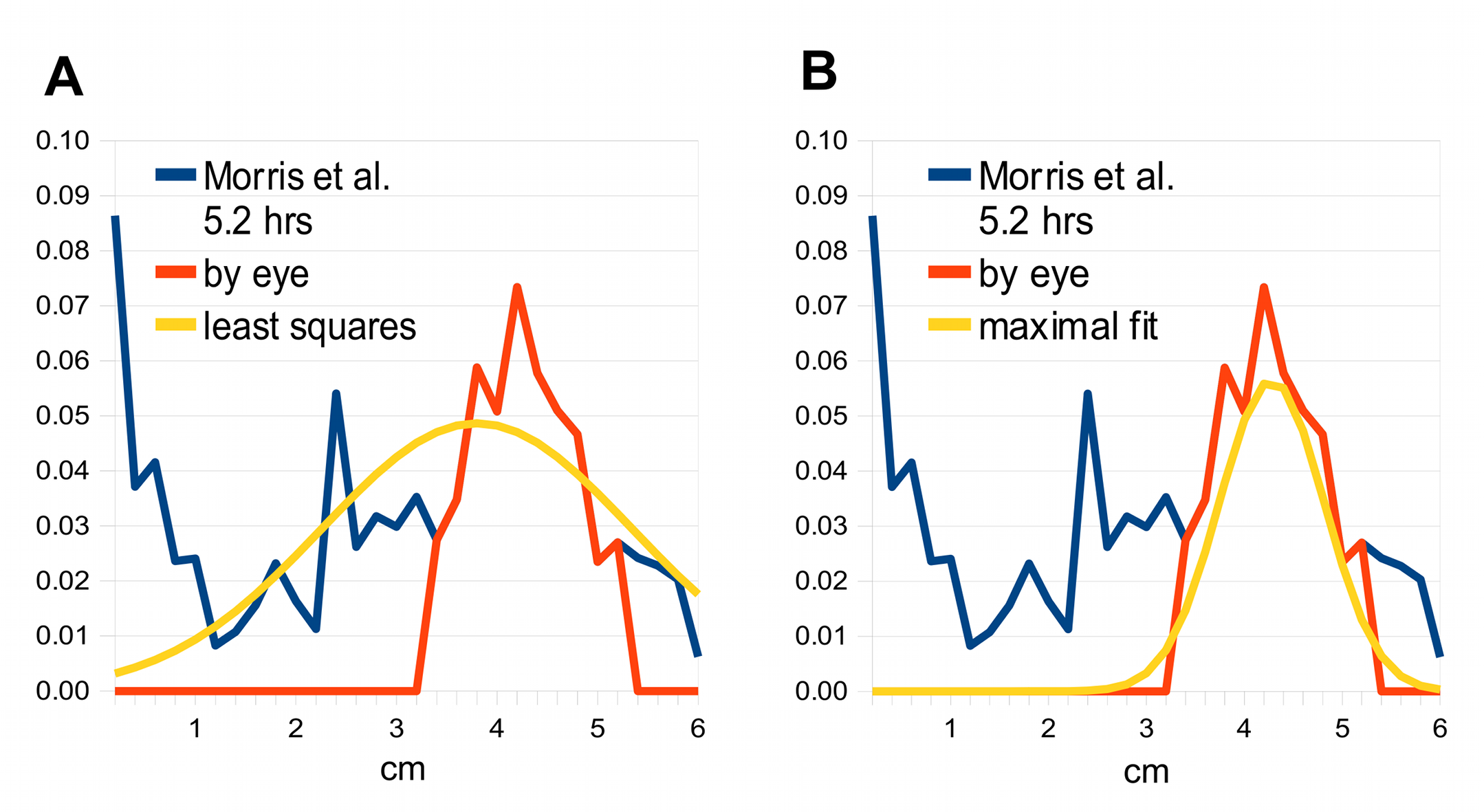

Supplement: S2 Fig — Fig 4 in Morris et al. [15] shows pulses that are the average from a number of segments. Here a pulse from an individual segment is shown (data kindly supplied by Dr Morris), revealing more noise than in the averaged data. A: Least squares compared with selection by eye. B: maximal fit (S1 Text) compared with selection by eye, showing much better agreement. (TIF) [file pcbi.1004487.s009.tif]

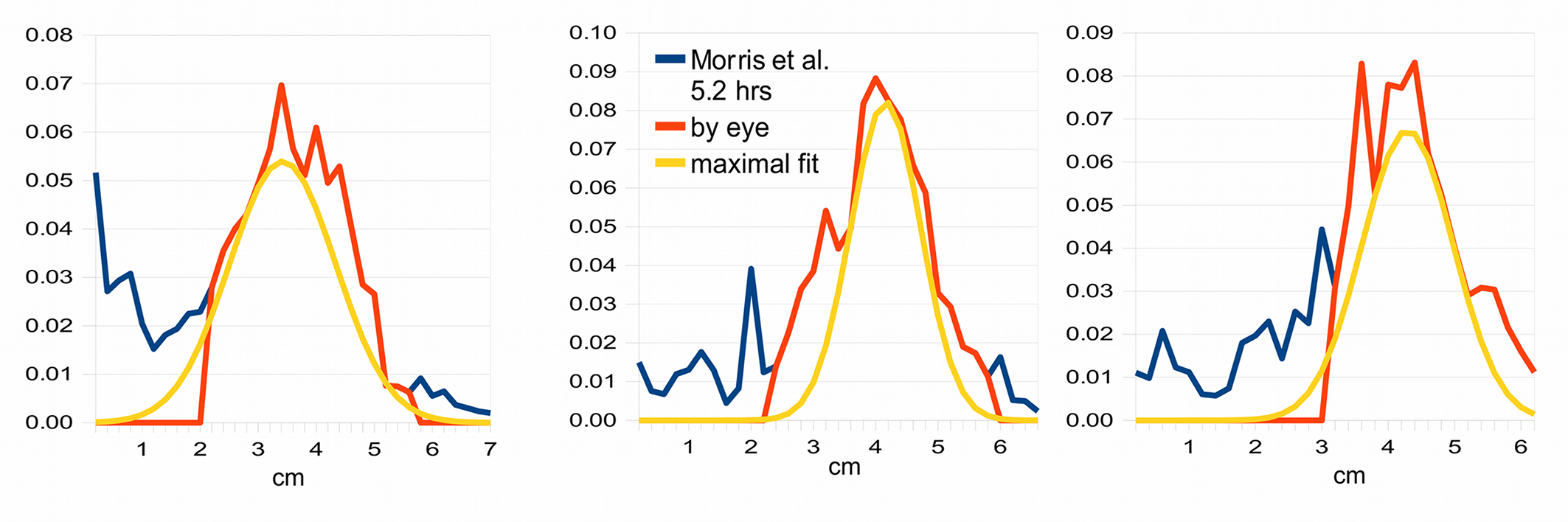

Supplement: S3 Fig — Reasonable concordance between the peak fitted by eye and the maximal fit algorithm (S1 Text): individual segment data for 5.2 hours underlying the averaged graph in Fig 4 in Morris et al. [15]. (TIF) [file pcbi.1004487.s010.tif]

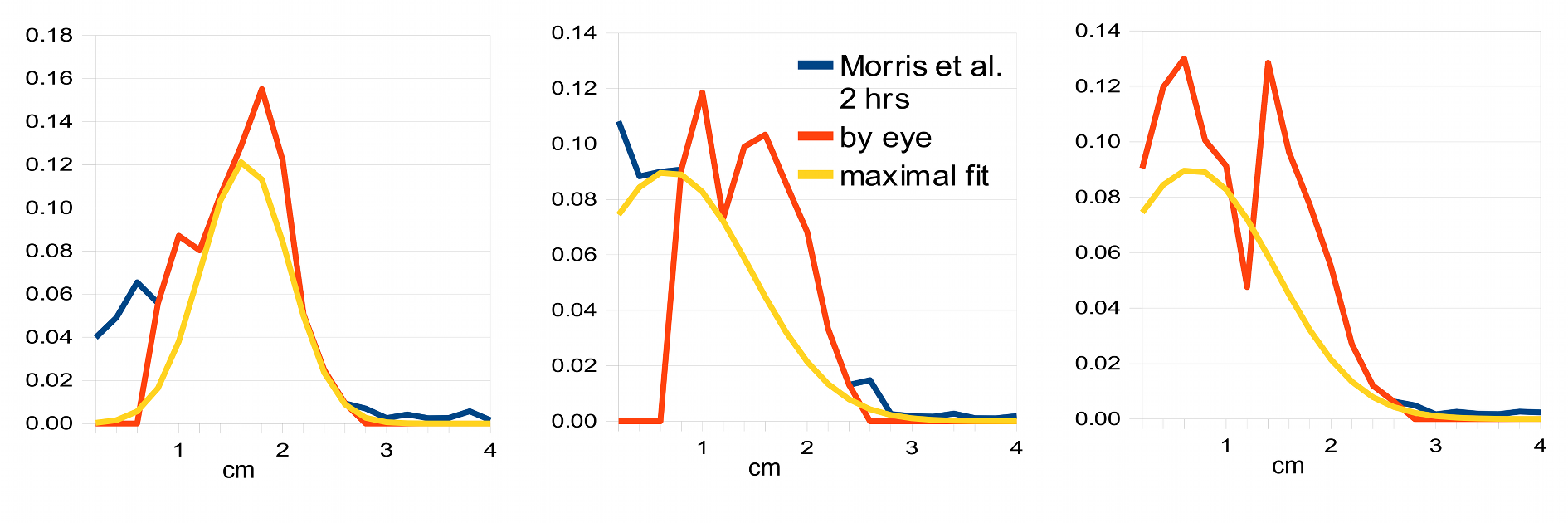

Supplement: S4 Fig — Less good agreement between eye and maximal fit for 2 hour individual segment data from [15]. (TIF) [file pcbi.1004487.s011.tif]

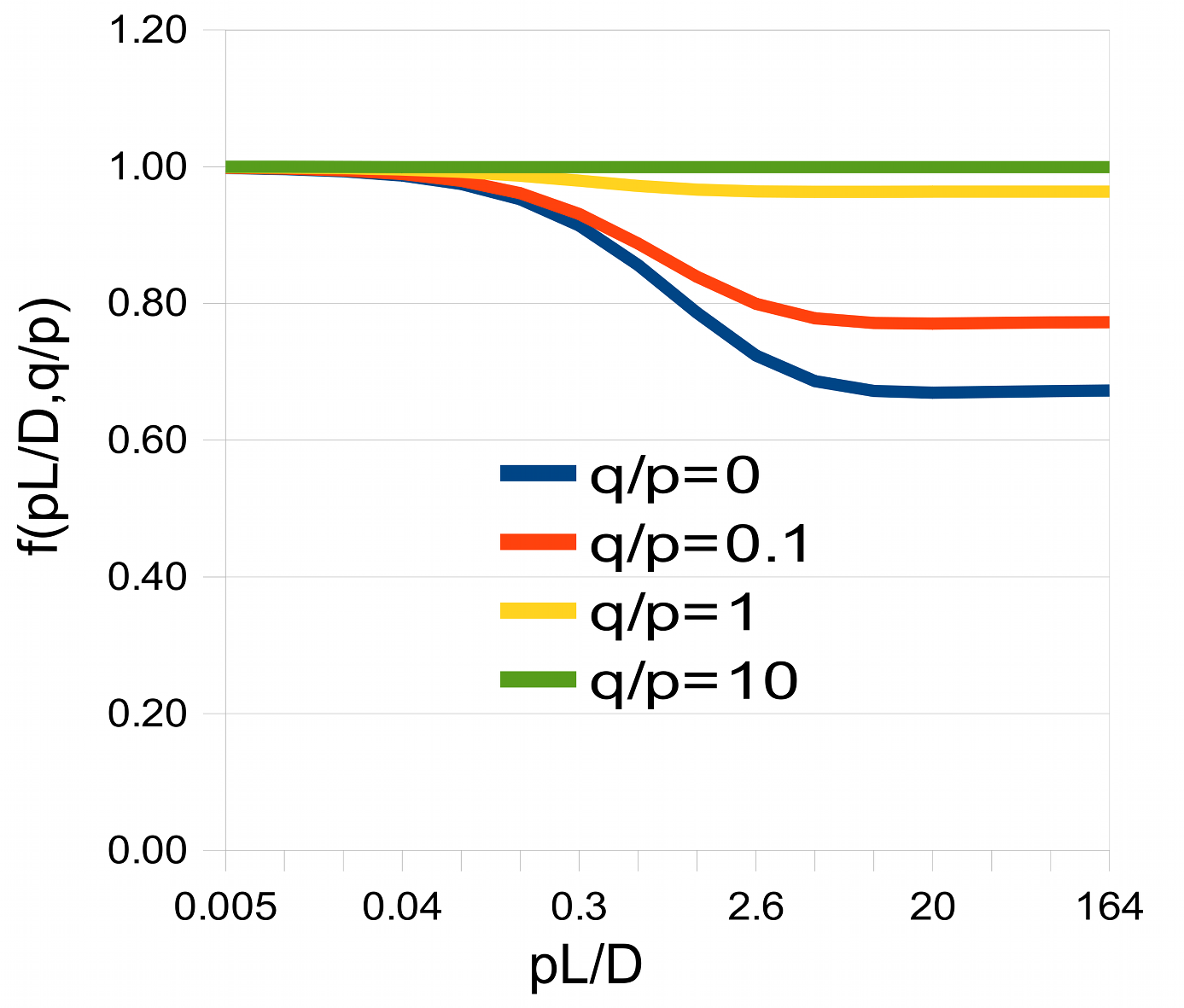

Supplement: S5 Fig — The function f(pL/D,q/p), with pL/D on the x-axis, and for various values of q/p; see S3 Text. For the simulations, the number of intracellular compartments was taken to be N = 10 (see Models). A pulse was initiated in a single compartment and allowed to progress down the stem for 30 minutes, its variance measured, and then, after a further 30 minutes of transport, measured again, thus allowing ρ to be estimated accurately. The function f was then calculated from Eq (S8). (TIF) [file pcbi.1004487.s012.tif]

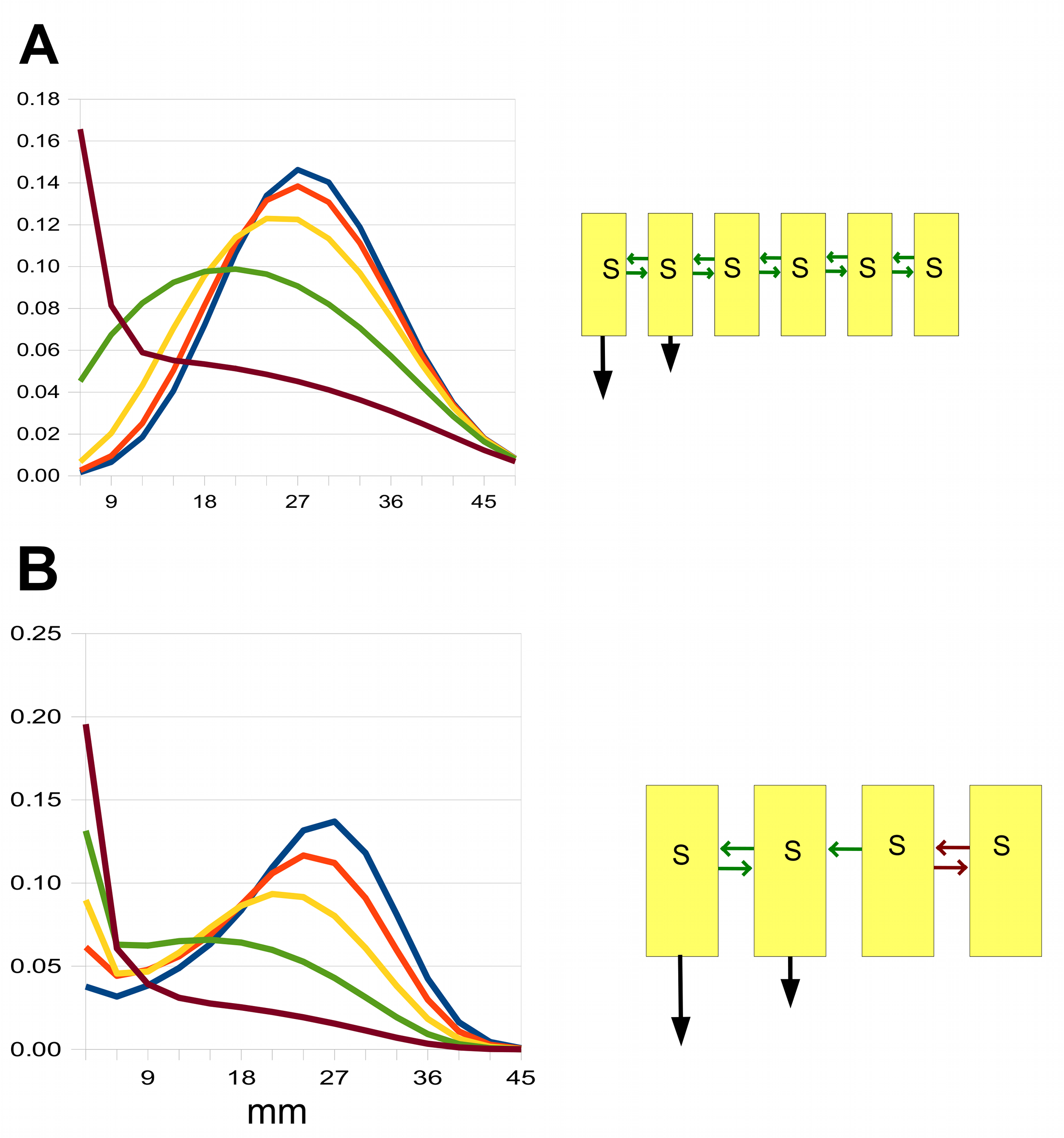

Supplement: S6 Fig — See S6 Text. A: First attempt at modelling the data in Fig 4A-4E in Brewer et al. [50] using the 6-channel model of Fig 6, with equal lateral permeability between all channels, i.e. without a weaker coupling between channels 5 and 6 as in Fig 10. B: A four-channel model that also produces a good fit to the data. Note the absence of an arrow from channel 2 to channel 3. In this model, the (unsaturated) permeabilities are p = 8 × 10−4 cm/sec in channel 1, p = 10−4 cm/sec in channel 2, and for channels 3 to 6 p = 0 and q = 2 × 10−3 cm/sec. The lateral, saturable, permeabilities have K m = 10−4, and s = 2 × 10−6 cm/sec for the green arrows and 5 × 10−7 cm/sec for the red arrows. For all channels, D = 3 × 10^−6^cm2/sec. (TIF) [file pcbi.1004487.s013.tif]
